# Determinants of Heterogeneity, Excitation and Conduction in the Sinoatrial Node: A Model Study

**DOI:** 10.1371/journal.pcbi.1001041

**Published:** 2010-12-23

**Authors:** Ronit V. Oren, Colleen E. Clancy

**Affiliations:** 1 Department of Physiology and Biophysics, Weill Medical College of Cornell University, New York, New York, United States of America; 2 Department of Pharmacology, University of California, Davis, Davis, California, United States of America; University of Calgary, Canada

## Abstract

The sinoatrial node (SAN) is a complex structure that exhibits anatomical and functional heterogeneity which may depend on: 1) The existence of distinct cell populations, 2) electrotonic influences of the surrounding atrium, 3) the presence of a high density of fibroblasts, and 4) atrial cells intermingled within the SAN. Our goal was to utilize a computer model to predict critical determinants and modulators of excitation and conduction in the SAN. We built a theoretical “non-uniform” model composed of distinct central and peripheral SAN cells and a “uniform” model containing only central cells connected to the atrium. We tested the effects of coupling strength between SAN cells in the models, as well as the effects of fibroblasts and interspersed atrial cells. Although we could simulate single cell experimental data supporting the “multiple cell type” hypothesis, 2D “non-uniform” models did not simulate expected tissue behavior, such as central pacemaking. When we considered the atrial effects alone in a simple homogeneous “uniform” model, central pacemaking initiation and impulse propagation in simulations were consistent with experiments. Introduction of fibroblasts in our simulated tissue resulted in various effects depending on the density, distribution, and fibroblast-myocyte coupling strength. Incorporation of atrial cells in our simulated SAN tissue had little effect on SAN electrophysiology. Our tissue model simulations suggest atrial electrotonic effects as plausible to account for SAN heterogeneity, sequence, and rate of propagation. Fibroblasts can act as obstacles, current sinks or shunts to conduction in the SAN depending on their orientation, density, and coupling.

## Author Summary

It is well known that a small structure in the atrium called the sinoatrial node (SAN) is the pacemaker for the heart. However, the complexity and heterogeneity intrinsic to this structure has made it difficult to determine some aspects of sinoatrial node function. Here we use a computational approach, based on experimental data, to tease out the individual contributions of cellular and tissue heterogeneities and the effect of fibroblasts and atrial cells on sinoatrial node function. The computational models suggest that the complex features of the intact sinoatrial node can be reconstructed with a relatively simple model. Our simulations also predict that the presence of non-cardiac cells in the node likely contribute to its function.

## Introduction

The sinoatrial node (SAN) is a complex heterogeneous tissue and its function may depend on this complexity [1]. Measurements from intact rabbit SAN have shown heterogeneity of electrophysiological properties from the center to the border of the atrium including gradual morphological changes in action potentials (AP), a decrease in maximum diastolic potential (MDP), an increase in peak overshoot potential (POP), an increase in upstroke velocity (UV) and a decrease in pacemaker potential slope [2], [3].

Some studies of the SAN describe a discrete-region model of SAN organization [4], comprising a central region of small primary pacemaker cells surrounded by a zone of larger transitional cells. Kodama et al. [5] observed AP variability in small balls of tissue isolated from SAN, and suggested a transition in ion channel expression as the cause. An additional series of articles in rabbit have reported that AP characteristics, current density, Ca^2+^ handling and connexin density are cell-size dependent [1], [6], [7].

More recently, Lyashkov et al. [8] identified three morphologically distinct SAN cells. However, experiments on enzymatically dissociated cells of all three types revealed *no* significant variations in APs, cycle length (CL), Ca2+ cycling or channel expression. Other studies have also failed to detect size-dependent (i.e. cell type dependent) differences in isolated SAN cells [9], [10].

From these disparate camps, two distinct hypotheses have arisen to explain intact SAN heterogeneity. The first is that the SAN has two specific cell types, central cells and peripheral cells, each with distinct electrophysiological characteristics [1], [6]. The second hypothesis suggests that all observed heterogeneity in the intact SAN results from electrotonic coupling effects - cells in the SAN near the atria will be strongly affected and modified by the atrium. Here we used a computational modeling approach to build distinct models based on the existing contrasting data sets that support the two hypotheses, and attempted to simulate experimentally measured properties of isolated SAN cells *and* characteristics of intact SAN tissue.

We then used the computational model to probe other anatomical factors that likely contribute to the observed function and heterogenetity in the SAN. It has been observed that fibroblasts constitute a larger fraction of the SAN, than atrial or ventricular tissue [11]. Anatomical studies of the rabbit SAN suggest a disorganized “mesh” of SAN cells arranged around “islands” of fibroblasts [11]. Fibroblasts form functional gap junctions with myocytes *in vitro* and *in vivo* [11],[12], [13]. Recent *in vitro* experiments suggest that electrically coupled fibroblasts alter impulse SAN conduction [12], [13] and that fibroblasts may affect the spontaneous activity of the SAN cells [14]. Fibroblast density increases with age and may play a role in ageing-induced bradycardia or sick sinus syndrome [15], [16].

Atrial cells are also dispersed throughout the SAN [11], [17] and it is unclear how these cells may influence SAN excitability. Verheijck et al. [11], [17] hypothesized that the gradual increase in density of atrial cells from the SAN center toward the atria causes a gradual increase in atrial electrotonic influence that underlies the transition from nodal to atrial action potentials.

Our computational modeling approach allowed us to examine how sources of heterogeneity in the SAN including cellular differences, gradients in coupling, fibroblasts and atrial myocytes in the SAN affect excitation and conduction. Our tissue simulations suggest electrotonic effects as plausible to account for SAN heterogeneity. Uncoupled fibroblasts act as obstacles to conduction in the SAN and, when coupled slow conduction by acting as current sinks, or shunt electrical activity between regions, depending on the orientation, density and coupling strength. Our model simulations also revealed only minor effects of atrial cells in the SAN.

## Methods

The Kurata rabbit SAN central cell model [18] was used as the base model for cell and tissue simulations in this study because it incorporates: 1) intracellular Ca^2+^ dynamics and a subsarcolemmal Ca^2+^ diffusion compartment, 2) the novel pacemaker current *I_st_*, 3) Ca^2+^ dependent inactivation of L-type Ca^2+^ channel 4) accurate activation kinetics of *I_Kr_*, 5) revised kinetic formulations for 4-AP-sensitive currents (*I_to_* and *I_sus_*). The Kurata model reproduces AP waveforms, ionic currents, effects of ion channels blockers and differential effects of BAPTA and EGTA on pacemaker frequency [18]. Please refer to Text S1 for details.

Effects of vagal stimulation were incorporated into the model in order to investigate the experimentally observed pacemaker shift in response to ACh application as we have done previously [19]. The concentration of ACh was chosen to approximate the experimentally observed effect of ACh on SAN cells, where maximum diastolic potential is hyperpolarized by 10 mV [20]. This concentration (1×10^−5^ M) results in effects on I_Ca,L_ and I_f_, and I_K_. All tissue level simulations are described in full detail in the online Text S1.

We modeled the sick sinus syndrome associated P1298L Na+ channel mutation, by modifying I_Na_ properties according to experimental data [21]. We incorporated a 13.5% negative shift of the inactivation curve and implemented the same percentage changes of the fast and slow inactivation time constants (τ_fast_ increased by a factor of 2, τ_slow_ increased by a factor of 3.6) as observed experimentally. The maximal Na^+^ channel conductance was reduced by 44% to reproduce the reduction in I_Na_ as recorded experimentally.

Simulations were carried out with a passive electrophysiological model of atrial fibroblasts as described previously [22]. Details are in Text S1.

Simulations were encoded in C/C++ and run on a Sun Fire X4440 ×64 Server and multiple Apple Intel based Mac Pros 3.0 GHz 8-Core using OpenMP with the Intel ICC compiler version 11.1. Numerical results were visualized using MATLAB R2009a by The Math Works, Inc. An explicit Euler method with a time step of 0.01 ms and Neumann no flux boundary conditions are used. All source code used in this paper is available by request to ceclancy@ucdavis.edu.

## Results

### Effect of coupling on pacemaker site location

**Figure 1:**
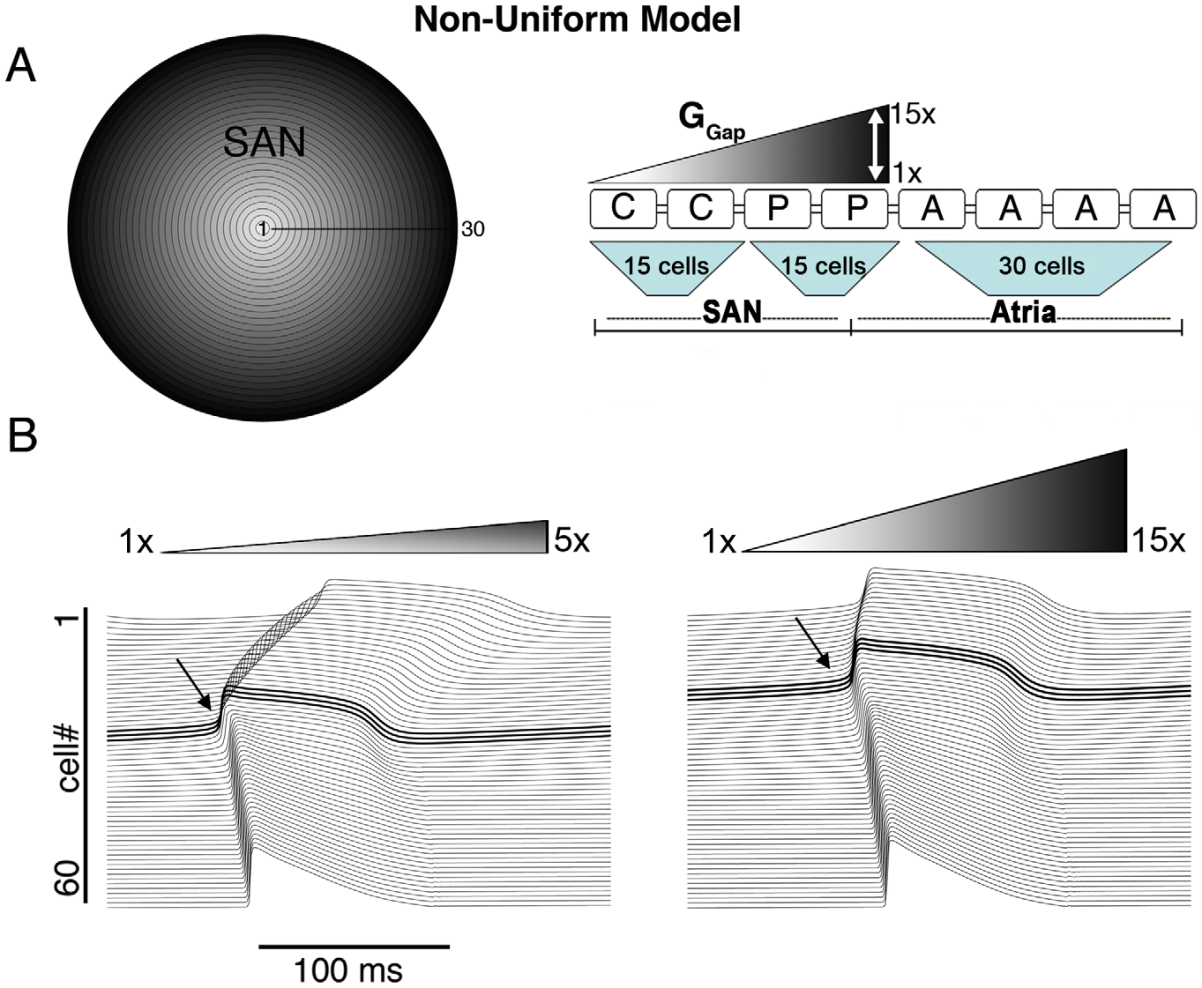
The non-uniform model. A) The non-uniform model schematic and its equivalent 1D strand with various levels of gradients in coupling is shown (‘C’ central cell, ‘P’ peripheral cell, ‘A’ atrial cell and ‘G_Gap_’ intercellular coupling). B) The site of pacemaker initiation (bold and arrow) under conditions of shallow (left) and steep (right) G_Gap_ gradient (respectively).

First, we constructed a “non-uniform” radially symmetric tissue model (Figure 1A), containing central and peripheral model cells based on experimental data that support this view (see Methods). We incorporated a linear gradient of cell-to-cell coupling from the center to the periphery of the SAN based on histological studies that show spatial gradients of gap junctions, resulting in a 10-fold increase in density from SAN center to periphery [2], [23]. A schematic is shown in Figure 1A, where low coupling is white and the gradient of increasing coupling toward the periphery is indicated by degree of shading.

**Figure 2:**
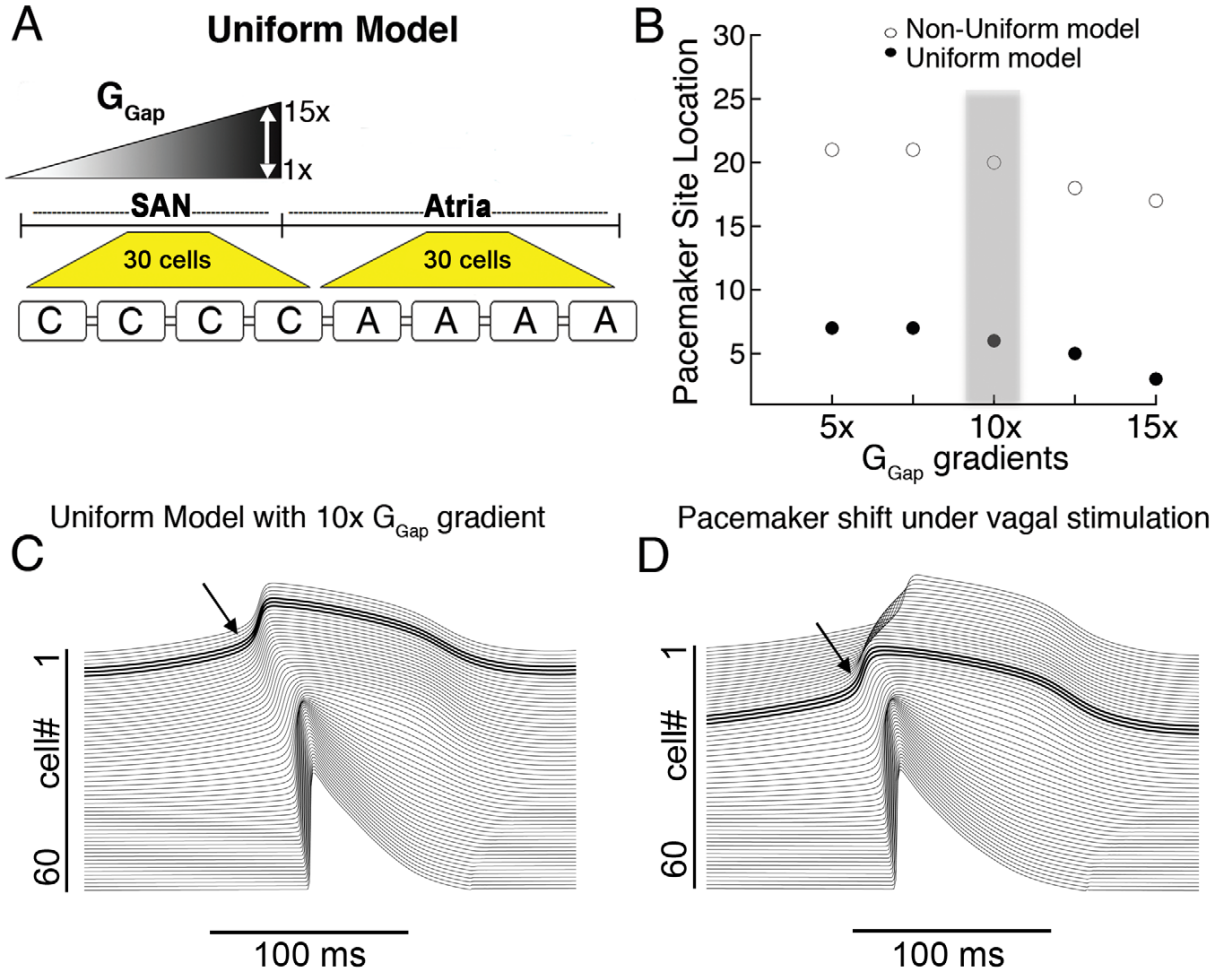
The uniform model. A) A schematic for the uniform model 1D strand is shown (‘C’ central cell, ‘A’ atrial cell and ‘G_Gap_’ intercellular coupling). B) Within the physiologically observed range of intercellular coupling, only peripheral pacemaker initiation occurs in the non-uniform model (open circles), while the uniform model simulates central pacemaking for a range of physiological coupling values. C) A 10× gradient is shown to initiate pacemaking in the center. D) A simulated shift of the pacemaker site to the periphery due to vagal stimulation (from cell #6 to cell #21, bold traces).

We tested a range of linear gradients spanning the experimental estimate (10-fold), from 5-fold to 15-fold [23]. We used the measured average intercellular coupling value [24] in the SAN center of 7.5nS (white). Figure 1B shows simulations of SAN excitation in the non-uniform model under conditions of shallow (from 7.5 nS in the center to 37.5 nS in periphery - 5-fold larger G_Gap_ in periphery, left) and steep (from 7.5 nS to 112.5 nS -15-fold larger G_Gap_, right) gradients. In both cases we observed initiation of pacemaking in the periphery – a result that is not consistent with experimental observations, where central pacemaking is observed (Figure 1B left, pacemaker site is bold). We were not completely surprised by this finding since in our single cell simulations the peripheral cell has a faster intrinsic excitation frequency (See Figure 2C in Text S1). We postulated that electrotonic coupling to the atrium might make peripheral cells less excitable by acting as a large current sink.

However, our simulations suggested this is not the case, and that no degree of “physiological” coupling shifted pacemaking to the SAN center in the non-uniform model (Figure 1B and Figure 2B, open circles). Moreover, the CL in the non-uniform model was 234 ms, which is markedly shorter than the experimentally observed values of 361±38 (see Table 1). The upstroke velocity (UV) of action potentials in the periphery (60.7 V/s) was too large and maximum diastolic potential (MDP) in the periphery was too negative and outside of the measured range (see Table 1).

**Table 1:** Comparison of intact SAN parameters.

	Uniform Model	Non-uniform model	Experimental Values
CL, ms	301	234	361±38
SACT, ms	13.9	2.7*	21±11
MDP (central SAN), mV	−57.6	−62.6	−56±11
MDP (peripheral SAN), mV	−68.6	−73.7	−61±6
APD_90_ (central SAN), ms	142.9	115	120–150
APD_90_ (peripheral SAN), ms	107	98	80–120
UV (central SAN), V/s	6.1	8.4	<10
UV (peripheral SAN), V/s	13.8	60.7	10–50

*A cell near the atrium fires first, resulting in very small SACT.

In order to be sure that the results presented above did not depend on the specific conductance values for each current, we performed a sensitivity analysis of pacemaker location to 10% perturbations (encompassing the experimental measurement ranges) in all ionic conductances. The pacemaker location was robust to increases or decreases in any conductance – peripheral pacemaking was always observed for the non-uniform model. The methods and results are contained in Text S1.

We next used the uniform model incorporating data that supports the hypothesis that ***no*** intrinsic differences exist between central and peripheral cells [8], [9], [10] and that observed differences in the intact SAN derive from proximity of cells to the atrium. This is supported by data in single cells where no differences in AP properties in large versus small cells isolated from the SAN were observed [8]. The 2D “uniform” model contains only central cells connected to the atrium (Figure 2A).

Figure 2B illustrates the pacemaker site for values of G_Gap_ gradients for the non-uniform (shown in Figure 1) and the uniform model. Electrical initiation in the first 15 cells is central pacemaking. Although the non-uniform model did not simulate central pacemaking for any values tested, the uniform model simulated central pacemaking over a wide range (Figure 2B, filled circles), beginning with a coupling gradient of 5×. Simulations are shown in Figure 2C using the uniform model with a G_Gap_ gradient of 10× [2], [23]. We performed the same sensitivity analysis as described above and found that central pacemaking was robust to changes in all conductances and always observed for the uniform model.

One of the important manifestations of the heterogeneous SAN is the shift of the pacemaker site in response to different innervations. In the case of vagal stimulation, the pacemaker shifts towards the periphery of the SAN [25]. Therefore, in order to mimic vagal stimulation in both our non-uniform and uniform models, we incorporated the recorded changes to ACh-activated K^+^ current (I_K,ACh_), I_f_ and I_Ca,L_ as we have done previously [19]. In the rabbit SAN, fine nerve processes form a basket around the pacemaker cells at the normal leading pacemaker site, but there are few or no visible fibers in the periphery of the SAN [26]. Following these data, we have applied the effects of vagal innervation only in SAN “central cells” (i.e. cells #1–15). Figure 2D shows that simulated vagal stimulation resulted in a shift in the pacemaker site in the uniform model to the periphery (cell #20) compared with control (Figure 2C, pacemaker site at cell #6), consistent with experiments. In the non-uniform model, on the other hand, simulated vagal stimulation resulted in a slight shift of the pacemaker site within the periphery of the SAN (from cell #19 in control to cell #22 under vagal stimulation, not shown).

The mechanism for the shift of the pacemaker site under vagal stimulation is as follows: During vagal stimulation, an increase in I_KACh_ results in a 10 mV hyperpolarization of the MDP in the central region of the SAN. ACh also partially inhibits I_Ca,L_, and shifts the activation curve of the hyperpolarization-activated current, If, towards more negative potentials. This leads to a slowing of diastolic depolarization and consequently pacing frequency. Cells in the periphery, which are unaffected by vagal stimulation have a faster intrinsic frequency and thus drive pacemaking.

### Effects of increased peripheral coupling

**Figure 3:**
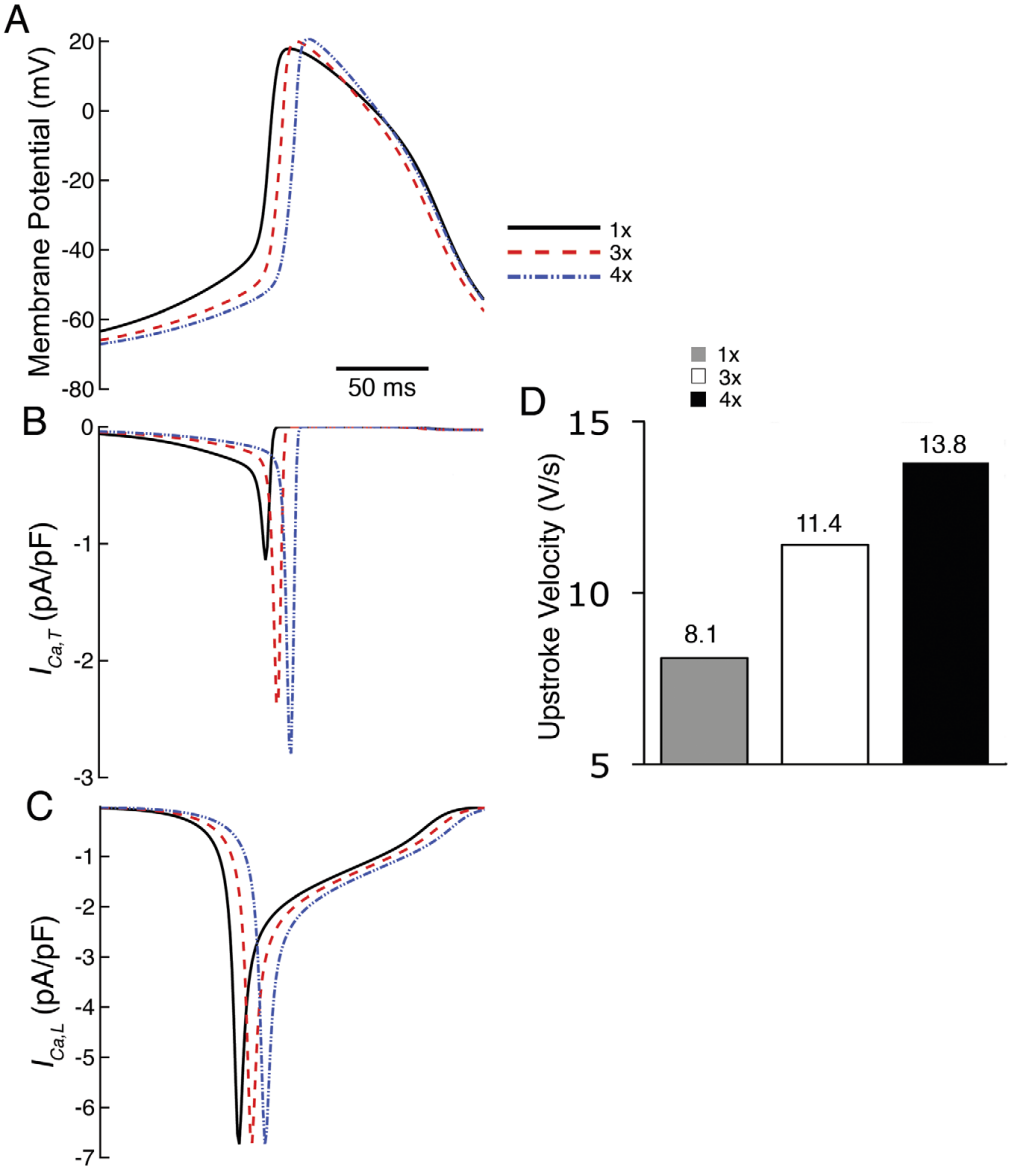
Effects of increased peripheral coupling on SAN propagation. A) APs and B) corresponding I_Ca,T_ and C) I_Ca,L_ computed at 3× and 4× increased peripheral coupling. D) Upstroke velocity increases with additional peripheral coupling.

Studies suggest that in addition to increased gap junction density in peripheral SAN, gap junctions in the SAN periphery are distinct isoforms with larger conductance [2], [23], [24], [27], [28], [29]. In the SAN center, Cx30.2, small conductance gap junctions form 30–40 pS channels with Cx40 and Cx45 [28]. In the periphery of the SAN 60–120 pS (2–4 fold increase in coupling compared to center) Cx43 and Cx45 are expressed [27], [30]. We thus assessed effects of increased peripheral coupling on the SAN by incorporating an additional multiplicative factor for peripheral coupling between 1 and 4 in cells #15–30. Additional peripheral coupling was applied to the uniform model with the 10× G_Gap_ gradient (from Figure 2C). Steady-state APs of a peripheral cell (cell #27) are shown in Figure 3A. As peripheral coupling was increased, peak overshoot potential (POP) increased (from 17.9mV in control to 20.9mV in 4× peripheral coupling), maximum diastolic potential (MDP) and take-of-potential (TOP) became more negative (from −66.7mV to −68.8mV and −44.1mV to −51.4mV) and diastolic depolarization (DD) slope was reduced (from 0.2mV/ms to 0.1mV/ms). Note also that action potential duration (APD) is reduced as peripheral coupling is increased, owing to the voltage dependent reduction in cellular input resistance in the neighboring atrium as rectification of I_K1_ currents is alleviated [31]. Figure 3D shows the effect of increased intercellular coupling in the periphery to reproduce observed increased upstroke velocity (UV) (cell #27) [1], [2].

Further investigation revealed that an increase in *I_Ca,T_* (from −1.14 pA/pF to −2.9 pA/pF) causes increased UV (Figure 3D). Increased peripheral coupling results in a reduction in DD slope and leads to lesser inactivation of *I_Ca,T_* , which increases the peak current of *I_Ca,T_* (due to more channel availability) during the TOP phase of the AP (Figure 3B). Although *I_Ca,L_* is the primary current activated during AP upstroke, it remained unchanged (Figure 3C) because the simulated decrease in DD primarily affects *I_Ca,T_* and not *I_Ca,L_*.

Central cells are not affected by the increase in peripheral coupling. Peripheral cells are increasingly affected according to their proximity to the atrium – those that are closest to the border of the atrium are most affected. This is because the increase in coupling “allows” cells in the periphery to “feel” more electrotonic influence of the atrium.

### Electrotonic modulation by fibroblasts

**Figure 4:**
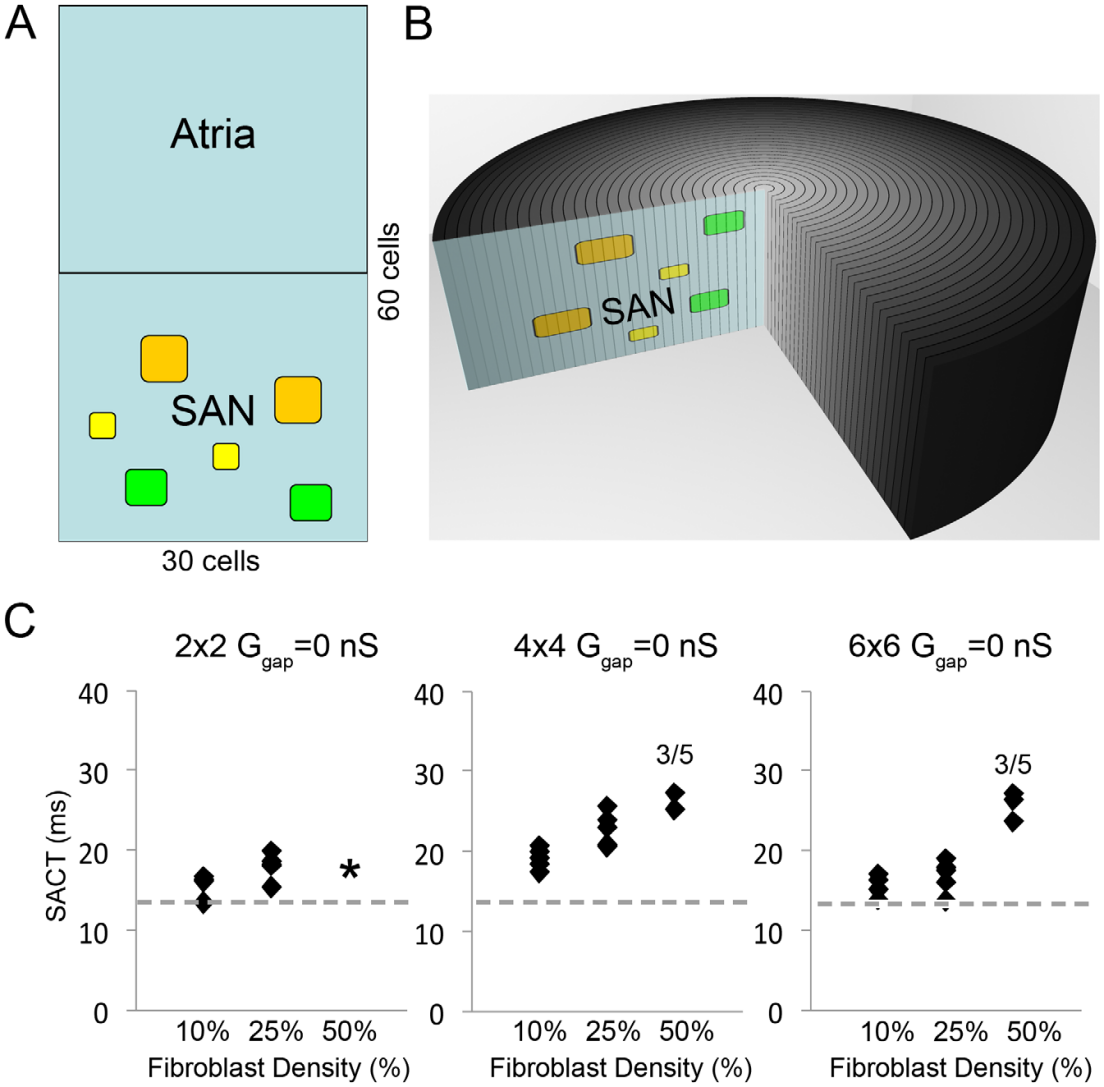
The effect of fibroblasts in the SAN. A and B: Schematic of a 3D tissue and 2D slice incorporating different sized islands of fibroblasts: 2×2 (yellow), 4×4 (green) and 6×6 (orange) within the SAN region. Panel C: Uncoupled fibroblasts and myocytes act as obstacles to electrical impulse propagation. For fibroblast-myocyte coupling of 0nS (G_gap_ = 0 nS), as fibroblasts percentage coverage increases sinoatrial node to atrium conduction time (SACT) increases for all islands sizes (left - 2×2, middle - 4×4, right - 6×6) and distributions (see Figure 3 in Text S1 for all distribution maps). Each fibroblast density (10%, 25% or 50%) is represented by 5 distributions (black diamonds). The dashed gray line denotes the SACT control value (13.8 ms).

Electrotonic coupling of cardiac myocytes and fibroblasts has been observed *in vitro* and *in vivo* in the rabbit SAN [11], [12], [13]. SAN tissue has fibroblasts interspersed in islands that occupy about 50% of SAN volume [32]. Because atrial fibroblasts are distinct from ventricular fibroblasts and lack large K^+^ currents observed in ventricular fibroblasts [14], [33], we incorporated an atrial fibroblast in the model [14] (schematic in Figure 4A and B), which allowed us to test the effects of fibroblast density (10%, 25% and 50% of the SAN), fibroblast island size (2×2, 4×4 and 6×6, Figure 4A) and the distribution of fibroblasts. Fibroblast distributions assumed no fibroblast island touched another island (as this would change the island size).

We first tested fibroblasts as obstacles to electrical propagation with various distributions and densities of **uncoupled** fibroblasts (G_Gap_ = 0 nS) and observed slowed conduction (SAN to atrium conduction time (SACT)) as a function of fibroblast density (Figure 4C). Neither island distribution (filled squares in each column denote five different fibroblast distributions as shown in Figure 3 in Text S1) nor fibroblast island size (islands composed of 2×2 (small), 4×4 (medium) and 6×6 (large) fibroblasts are shown as indicated in Figure 4C, respectively) largely affected SACT. 50% fibroblast density with 2×2 islands could not be obtained within the constraint that islands not touch islands (asterisk in panel A). With large plentiful fibroblast islands (6×6) and clustered near the atrium (50% fibroblast density in right panel), the atrium failed to excite (Figure 4C) (see Figure 3 in Text S1 for fibroblast distributions – here, distributions 3 and 5 failed to excite the atrium). A fibroblast barrier near the atrium caused a mismatch between availability of depolarizing charge (source, the SAN) and charge required for excitation (sink, the atrium). The intrinsic excitation frequency of the SAN was unaffected by the presence of fibroblasts (302ms) in all simulations at G_Gap_ = 0 nS (not shown).

**Figure 5:**
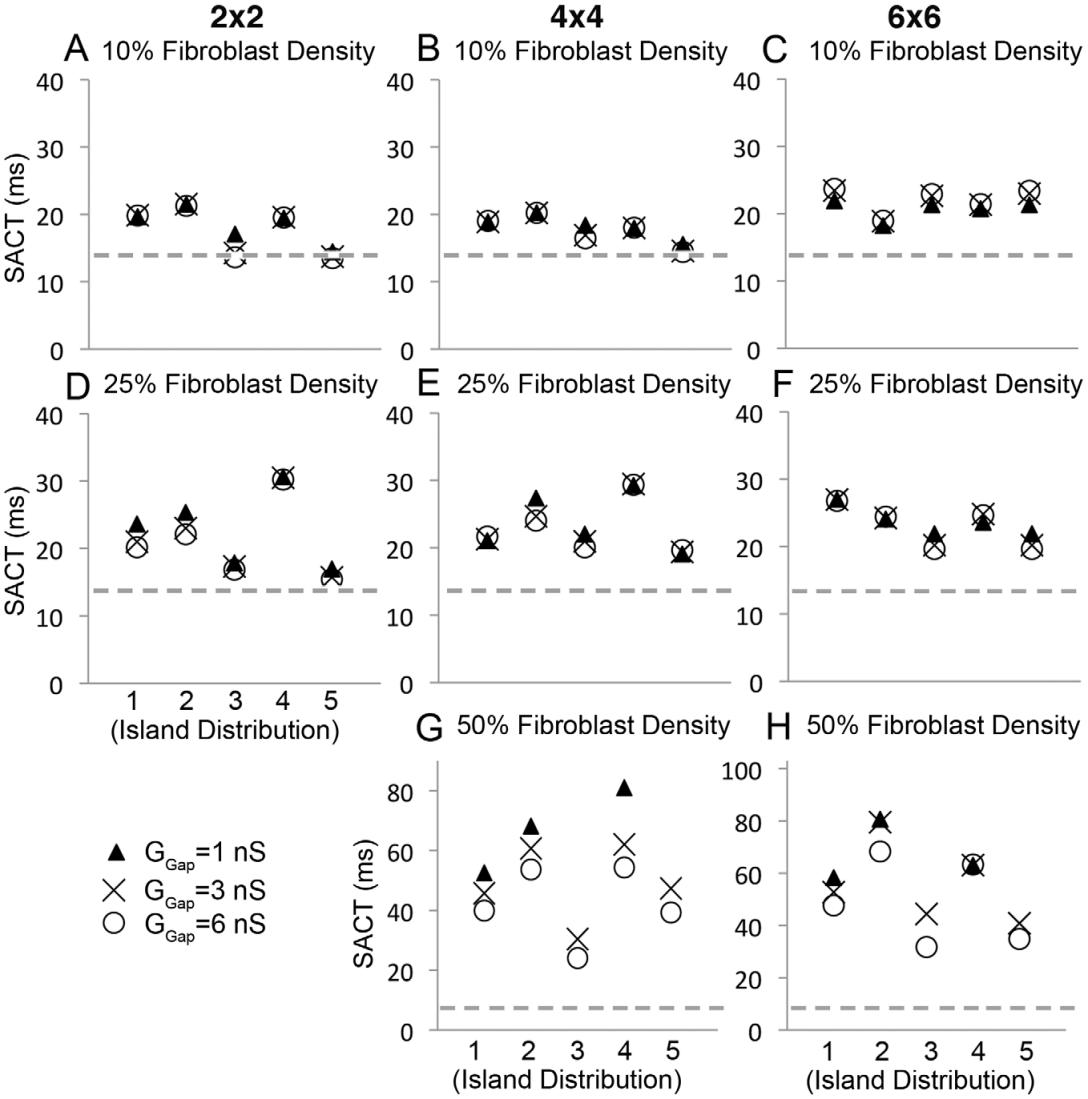
Modulation of sinoatrial node to atrium conduction time (SACT) by fibroblasts is shown. Panels show effects of different fibroblast spatial distributions, island sizes, fibroblast density and fibroblast-myocyte coupling values (G_gap_). In each panel, G_gap_ is 1 nS (triangle), 3 nS (X) or 6 nS (circle). A–C) 10% coverage - neither island distribution nor coupling affect SACT. D–F) 25% coverage - island distribution does affect SACT (see text). G–H) 50% coverage - both island distribution and coupling affect SACT. The dashed gray line denotes the value of SACT (13.8 ms) without fibroblasts.

Next, we explored the effect of the strength of coupling between myocytes and fibroblasts on impulse propagation in the SAN by setting G_Gap_ between myocytes and fibroblasts to 1, 3 and 6 nS, from the measured myocyte-fibroblast coupling range [34]. We simultaneously varied fibroblast density, island size and island distribution (Figure 5). As observed experimentally [12], [13], [35], our simulations predict fibroblasts induced slowing of conduction and increased fibroblast density increased SACT. With a fibroblast density of 10%, conduction was slowed, but additionally varying island size (compare panels A, B and C), island distribution (compare columns in individual panels) or coupling between myocytes and fibroblasts (note clustering of symbols) did not have large additional effects on SACT (Figure 5, A–C). However, when we increased fibroblast density to 25% (Figure 5, D–F), island distribution affected conduction time (SACT). In particular, distributions #3 and #5, where islands of fibroblasts were clustered near the edges of the SAN, sped conduction, while distribution #4 with centralized islands slowed conduction.

Increasing fibroblast density to 50% revealed how coupling (G_Gap_) affects conduction (Figure 5, G–H). In most distributions with 50% fibroblast density, when G_Gap_ is large (6 nS, circle) SACT is shorter (compared to G_Gap_ = 3 nS, denoted by “X” or 1 ns, filled triangle). Electrical impulses pass through a tightly coupled fibroblast island faster than when weakly coupling. As in the uncoupled simulations (for G_Gap_ = 0 nS, Figure 5C), distributions #3 and #5 (see Figure 3 in Text S1) failed to excite the atrium for weak coupling (G_Gap_ = 1 nS, Figure 5H). Distributions with more fibroblasts at the border of the atrium fail to allow sufficient current to pass for excitation of neighboring atrial cells.

**Figure 6:**
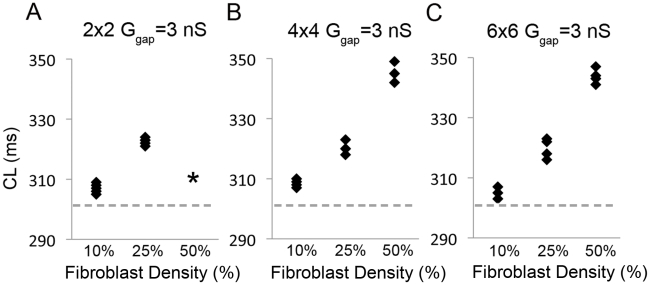
Excitation frequency decreases as fibroblasts density increases for all fibroblast island sizes (A - 2×2, B - 4×4, C - 6×6). The density (10%, 25% or 50%) for 5 fibroblast distributions (black diamonds) with G_gap_ = 3 nS. The dashed gray line denotes the CL control value (302 ms).

Figure 6 shows the impact of fibroblast density alone on excitation frequency (cycle length - CL). CLs at G_gap_ = 3 nS for 2×2, 3×3 and 4×4 size islands and 10%, 25% and 50% fibroblast density are shown. As fibroblast density increased, CL increased. This simulation may explain the reduced frequency observed in the SAN of ageing and diseased hearts [15], [16].

### Effect of interspersed atrial cells in the SAN

**Figure 7:**
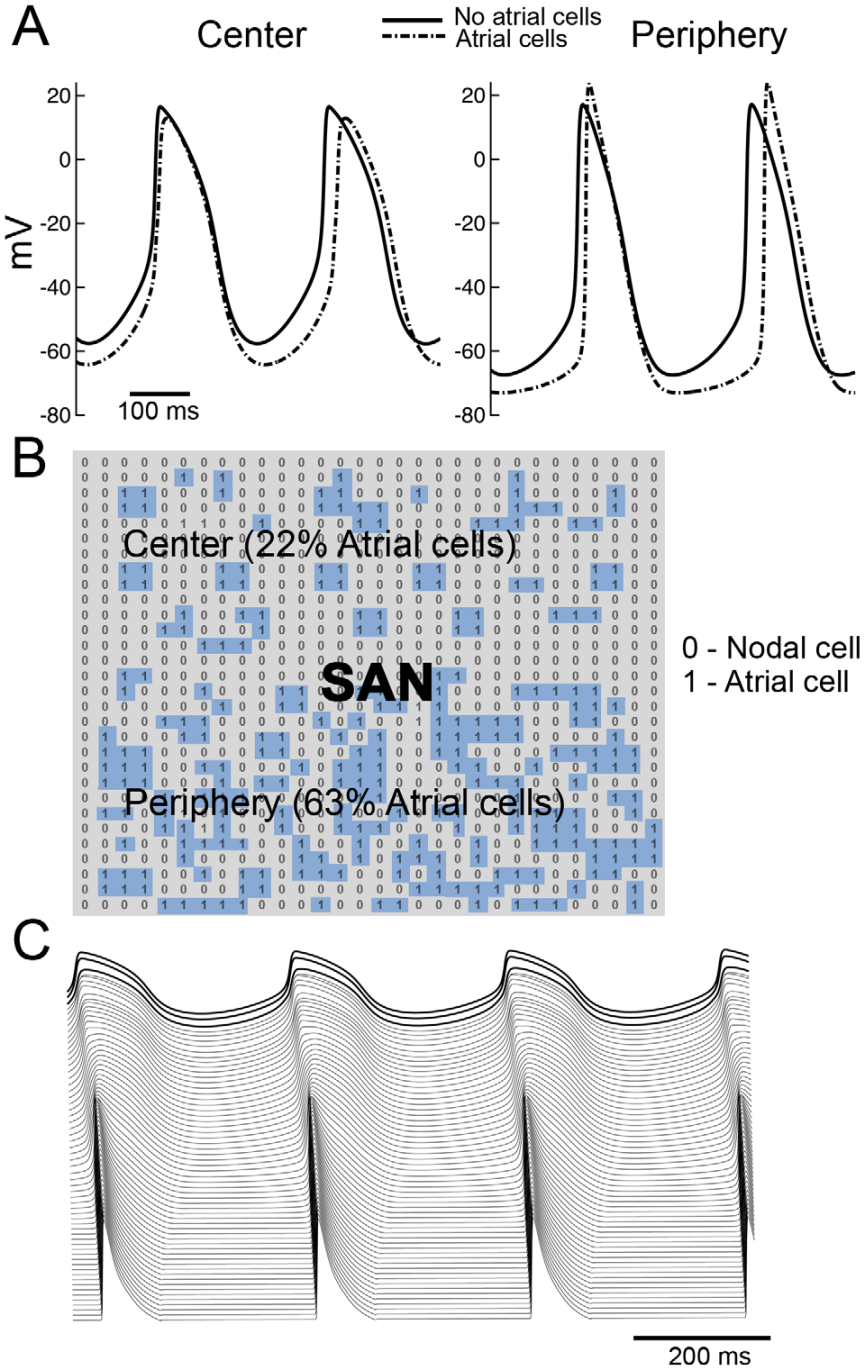
Atrial cells interspersed through the sinaoatrial node have little effect on SAN properties. A) Simulated APs in the presence (dotted line) and in the absence (solid line) of atrial cells. B) The matrix showing spatial organization of atrial cells in the SAN, where “1” denotes atrial cell (blue) and “0” sinoatrial nodal cell. The SAN center contains 22% atrial cells, which gradually increases to 63% in the periphery [10]. C) A space-time plot showing initiation of action potentials in the SAN center and propagation to the atrium in a mosaic model representation.

Verheijck E. et al. [10] have observed atrial cells interspersed in the SAN and suggested a “mosaic” model of SAN and atrial cells for SAN organization. In the mosaic model, the percentage of atrial cells varies from 63% in the periphery to 22% in the center. We incorporated a random distribution of atrial cells with a density of 22% in the center increasing gradually to 63% in the periphery [10] (schematic in Figure 7B, atrial cells in blue) connected via G_gap_ as in the neighboring nodal cells (there is no evidence for atrial specific gap junction channel (Cx43) presence in the SAN). Our simulations suggest that atrial cells have minor effects on electrical properties and behavior of the SAN (Figure 7A). CL and SACT in our mosaic model are longer (322 ms, 32.6 ms, respectively) compared with control (302 ms, 13.9 ms) because MDP is more hyperpolarized when atrial cells are present in the SAN. UV in the center is slower in the mosaic (4.2 mV/ms compared with 6 mV/ms in control). In contrast, UV is faster in the periphery (18 mV/ms compared with 13.8 mV/ms), consistent with experimental observations (see Table 1 in Text S1). Interspersed atrial cells also shorten APD in both central and peripheral regions of the SAN owing to the influence of I_K1_ – repolarization alleviates I_K1_ rectification leading to reduced resistivity, which increases the electrotonic influence of the atria during repolarization and during the diastolic interval (see also Table 1 in Text S1). Figure 7C shows the propagation of an impulse generated in the central region of the SAN through a peripheral region of increased coupling (as described in Figure 2) into the atrium as generated by the mosaic model. A simulation showing the effects of vagal stimulation in the central region (first 15 cells) of the mosaic model is shown in Figure 5 in Text S1. Consistent with our previous results, vagal stimulation in the mosaic model results in a peripheral shift of pacemaking and a slowing in CL.

**Figure 8:**
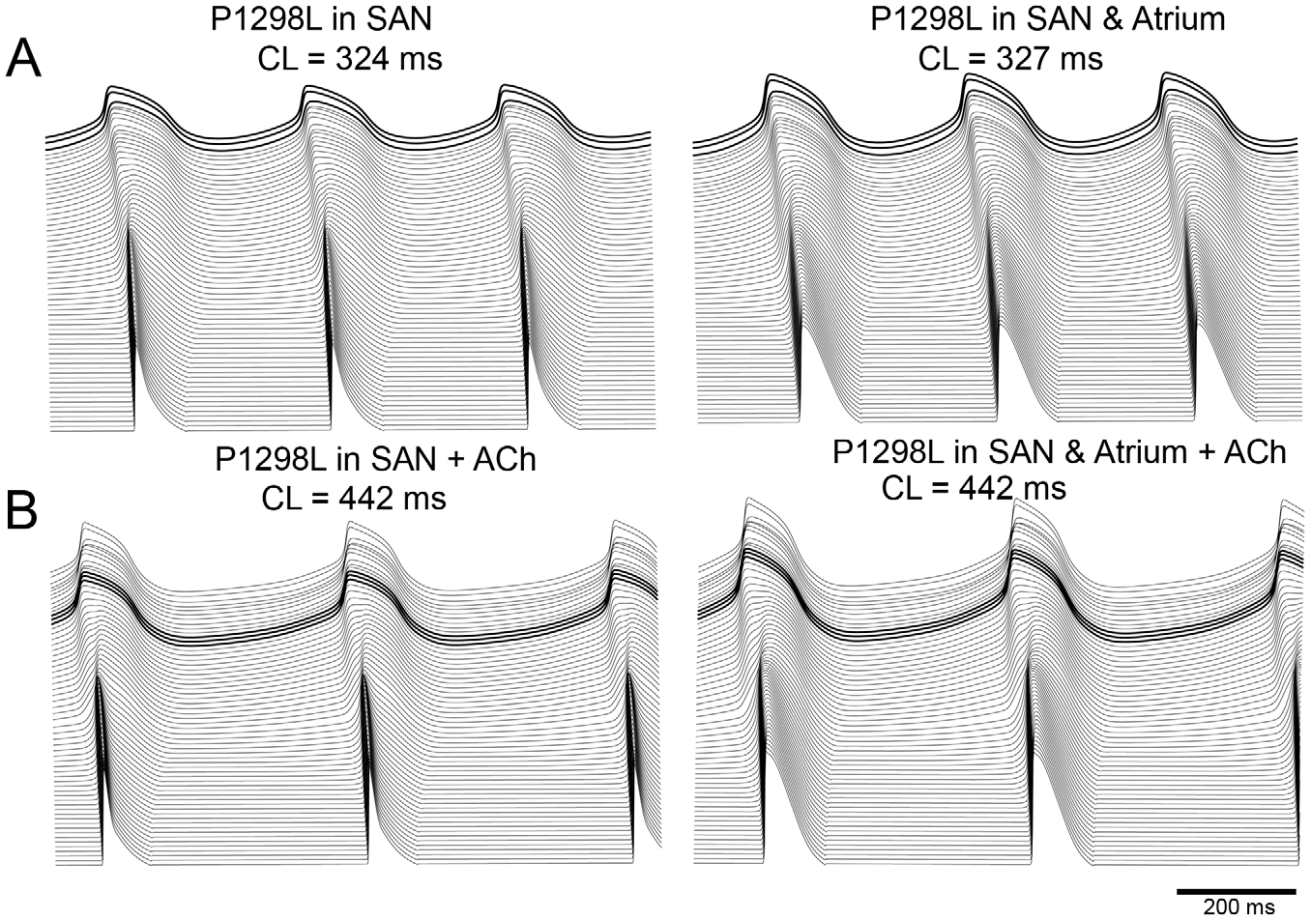
The effects of the familial sick sinus syndrome (SSS) Na+ channel mutation P1298L in the mosaic model. Figure 8A and B: Simulations after incorporation of the P1298L sodium mutation with and without vagal stimulation in the mosaic model are shown. The left panels of A and B show the effect of the mutation on interspersed atrial cells within the SAN alone. The right panels show the effect when the mutation is also incorporated into atrial cells.

Finally, we tested the effect of a familial sick sinus syndrome mutation that results in altered inactivation kinetics leading to a loss of Na^+^ current [21]. Figure 8 depicts the simulation predictions after incorporation of the P1298L sodium mutation with and without vagal stimulation in the mosaic model (Figure 8A and B, respectively). We observe mutation induced slowing of beating frequency and conduction velocity from the SAN to the atria, which becomes dramatic in the presence of ACh. Because no Na^+^ current is present in the SAN cells, the mutation effect results from the loss of Na^+^ channel function in atrial cells only. Interspersed atrial cells within the SAN that are affected by the mutation are less excitable and consequently draw more current from neighboring SAN cells (left panels of A and B, show effect of mutation on interspersed atrial cells within the SAN alone). This results in a slowing of pacemaking. Due to mutual entrainment in the SAN region we observed a slowing in heart rate in all cells. The presence of vagal stimulation dramatically exacerbates the mutation effects – by additionally slowing diastolic depolarization (and thus pacing frequency) in SAN cells (panel B of Figure 8). Note that when the mutation is also incorporated into atrial cells (right panels of A and B), atrial action potential duration is increased. This is a result of the mutation-induced reduction in the peak overshoot potential in the atrial cells (38.35 mV versus 24.29 mV with the mutation) that reduces the driving force of repolarizing K+ currents.

## Discussion

One goal of this study was to test two existing hypotheses for heterogeneity of the intact SAN. The first derives from a series of studies (reviewed by Boyett MR *et al.* [1]), which suggests the SAN is composed of central and peripheral cells that differ in intrinsic electrical properties [1], [6], [7], [18], [36], [37]. Our Kurata *et al.* [18] based central and peripheral cell models reproduced single cell experimental data supporting the “multiple cell type” hypothesis (Figure 1 and Table 1 in Text S1), but did not simulate observed tissue behavior. No values within the range of physiological coupling were sufficient to initiate pacemaking in the center of the SAN (Figure 2B).

The second hypothesis suggests that the SAN is composed of electrically uniform nodal cells [8], which is supported by data where no differences in APs, Ca^2+^ cycling or current densities were observed [8], [9], [10]. Thus, we generated a “uniform model” of only central cells connected to the atrium, which simulated central pacemaking initiation and impulse propagation (Figure 2B) consistent with experiments. Simulated behavior reproduced experimental parameters (see Table 1) (see also Table 2 in Text S1) and AP morphology (Figure 4 in Text S1). Our tissue simulations suggest atrial electrotonic effects as plausible to account for heterogeneity, activation sequence and rate of propagation of the SAN. Gap junctional coupling and inwardly rectifying properties of I_K1_ currents in the atrium generate a current sink, which depresses and delays depolarization of the periphery, allowing central cells (cells 1–15) to lead pacemaking.

From the center to periphery to atrium, AP upstroke velocity, peak overshoot potential (POP) and MDP increase, while APD and pacemaker potential slope are reduced [1]. The atrium delays the depolarization of the periphery by lengthening diastolic depolarization (reducing pacemaker potential slope) and lowering peripheral MDP, which alleviates inactivation of *I_Ca,T_* (Figure 3B) causing increased UV and POP. The decrease in APD along the conduction pathway is attributable to the shorter APDs in atrial cells, especially by alleviation of I_K1_ rectification as the cells repolarize, causing a repolarization gradient which “pulls down” plateaus of nearby cells leading to repolarization in the direction opposite to depolarization [31].

We used the model to address the role of fibroblasts in the SAN as they account for nearly 50% of the SAN cell density in rabbit [32]. Our simulations support evidence [35] that fibroblasts are a current sink by *hyperpolarizing* the SAN (as opposed to depolarizing neighboring myocytes in atrium and ventricles) causing a decrease in DD slope, an increase in CL and slower conduction. This is consistent with *in vitro* studies in spontaneously beating murine atrial cardiomyocytes [35] and in atrial [38] and ventricular [12] cells, where an increase in fibroblast density caused attenuation of spontaneous activity and increased conduction time (Figure 6). The study by Fahrenbach et al. [35] showed that the electrical interaction between fibroblasts and myocytes in co-cultured monolayers reduced frequency in a ratio-dependent but coupling-independent manner.

Our simulations results suggested three distinct parameter regimes: 1) the first is when fibroblasts were only barriers to conduction (G_gap_ = 0 nS), which caused an increase in SACT alone as fibroblast density increases. An increased fibroblast density provided the atrium less SAN cells (sources) to pull current from, thus enhancing the atrial loading effect and opposing depolarization in SAN cells. This manifested as a more hyperpolarized SAN cells with a longer DD phase that delays excitation. 2) When fibroblasts were coupled to SAN cells, there was an increase in both SACT and CL with increasing fibroblast density. Here, in addition to the decrease in electrical source for the atrium, the coupled fibroblasts acted as additional current sinks, which further reduced the DD slope and lengthened the DD phase. 3) At very high fibroblast density (50% or more), we observed an increase in SACT with low intercellular coupling (Figure 6 G–H). This is because electrical impulses readily pass through a tightly coupled fibroblast island - fibroblasts as electrical shunts.

The presence of atrial cells in the SAN region was also investigated. Unlike previous model studies [36], [39], [40] that did not support the mosaic model as a plausible model of SAN organization, our simulations suggest only modest effects of experimentally observed density of atrial cells on SAN function (Figure 7).

Finally, we investigated the effects of the familial sick sinus syndrome (SSS) mutation P1298L in our mosaic model and found that Na^+^ current need not be present in the sinus node to observe the reduction in pacing frequency and slowed conduction that are hallmarks of the disease [21]. Because no Na^+^ current is present in the sinoatrial node cells, the mutation effect results from the loss of Na^+^ channel function in atrial cells only. Interspersed atrial cells within the SAN that are affected by the mutation, are plagued by reduced excitability, causing them to act as a current sink as they require more current from the neighboring SAN to activate. This causes slowing of pacemaking and reduced conduction velocity of the impulse to the atrium. Due to mutual entrainment in the SAN region a slowing in pacemaker frequency is observed in all cells, which becomes much more dramatic in the presence of vagal stimulation. Sinoatrial node to atrial conduction time was also markedly increased with vagal stimulation.

Interestingly, despite different model representations, we observed very similar effects of the SSS mutation to those recently reported in the Butters et al. paper [41]. The mechanism as described in their study is also consistent – an increase in the current sink attributable to atrial cells - is the culprit.

### Comparison to previous model studies

When we began this study, we found that the widely used central and peripheral cell models reproduced measured single cell properties from experiments that supported the existence of two distinct cell populations [7]. But, in our hands, without a number of unjustified parameter modifications these cell models could not be coupled together and reproduce observed widely agreed upon tissue properties of the SAN, including central pacemaking [2].

Close inspection of the existing literature led us to suspect that a number of other groups that have attempted a heterogeneous reconstruction of the SAN with central and peripheral cells also failed. Zhang et al. [7] first developed mathematical models of central and peripheral SAN cells and a 1D gradient model of the SAN, which were subsequently modified by Garny et al. [37] in tissue simulations. The existing models that contain both cell types are rife [27], [37], [41] with parameter modifications (by starkly isolating peripheral cells and altering their conductance properties to make them more “central” like) to shift pacemaking to the center and “make it work”. These modifications of SAN single cell model parameters made them less representative of experimentally determined single cell activity.

When we considered that cellular properties from the SAN were more uniform than had been previously reported and built a model based on those data, we could reproduce measured cell AND tissue properties in the SAN without any parameter modification. We hope that this study will put to rest much of the “wiggly jiggly” with parameters that has been done with SAN tissue models over the years – we show that a simple model composed only of central cells can reproduce measured SAN properties – both in single cells and in tissue. Most importantly, our uniform model reproduced experimental tissue properties without arbitrary parameter modifications.

A very recent study by Butters et al. appears to be largely consistent with the results that we present here [41]. Although distinct “peripheral” pacemaking cells are included in their 2D SAN tissue model, these cells are present at very low density, surrounded by atrial cells or non-excitable regions (block zone), and do not appear to be present in the conduction pathway of the excitable impulse generated in the SAN center. In the Butters model, peripheral cells have apparently been “uncoupled” from the pacemaker by isolating very small low-density clusters within large atrial or inexcitable sinks. This seems consistent with our findings – a heterogeneous model of distinct central and peripheral cells will fail to evoke central pacemaking. We speculate that removing the peripheral cells from the simulation would have no effect on the results from the Butters paper.

In an early study to probe propagation in the SAN, Joyner and van Capelle [42] constructed a radially symmetric simplified 2D single cell type SAN model and adjoining atrial tissue. Their model predicted that driving the atrium required an SAN five times larger than reported [23]. In our model, SAN drove the atrium regardless of size (not shown). Joyner and van Capelle also reported that atrial excitation required partial uncoupling of the SAN from the atrium. Again, no such modifications were required with our model. Joyner and van Capelle [42] noted that a gradual increase in coupling allowed the SAN to successfully drive the atrium. Here, our simulations support the notion that an increase in intercellular coupling from center to periphery is a prerequisite.

### Limitations of this study

In future studies, different geometric configurations (such as the block zone, interdigitations between the SAN and atrial tissue border, interweaving cells in the center etc.) could be incorporated into the model. Although mechanosensitive currents in fibroblasts have been described in the SAN [22], the intrinsic feedback mechanisms controlling the currents are still controversial and so we did not apply descriptions of mechano-electrical feedback in our model fibroblasts. A very recent study in atrial fibroblasts observed TRPM7 channels that account for Ca^2+^ influx in atrial fibroblasts and are markedly upregulated in patients with atrial fibrillation where they may underlie pathological fibrosis [43]. This new finding is not accounted for in our study. Finally, in this study we have neglected the effects of sympathetic stimulation of the SAN. Clearly, sympathetic innervation will constitute an important component of future studies.

In conclusion, we showed that a relatively simple model is sufficient to represent many observed properties in the SAN. Our tissue model simulations suggest that the atrial load is a primary determinant of heterogeneity of the SAN, sequence and rate of propagation. In addition, we show fibroblasts as obstacles, current sinks, or shunts to SAN conduction depending on distribution, density and coupling.

## Supporting Information

Text S1 This file contains methods, figures, and tables.
(2.22 MB DOC)
