# Supplementary material for: Determinants of Heterogeneity, Excitation and Conduction in the Sinoatrial Node: A Model Study
Source: PLoS Comput Biol. 2010 Dec 23;6(12):e1001041. doi: 10.1371/journal.pcbi.1001041 (PMC3009599; doi:10.1371/journal.pcbi.1001041)
Supplement: Text S1 — This file contains methods, figures, and tables. (2.22 MB DOC) [file pcbi.1001041.s001.doc]

**Supplemental Methods:**

*Fitting normalized current densities in the peripheral cell model*

Based on studies^1-3^ in central and peripheral cells, we matched *I_Ca,L_*, *I_Na_,* *I_Kr_*, *I_Ks_* in peripheral cells of the "Non-Uniform Model" to normalized densities from experimental data. For the central cell model the normalized densities of *I_Ca,L_*, *I_Na_*, *I_Kr_*, *I_Ks_* at C_m_=32 pF are taken from the original Kurata et al.^4^ model. The density of *I_Na_* was set to -78 pA/pF (Figure S1 D, upper right star) in the peripheral cell model following the measured peak inward current at -5 mV in response to depolarizing voltage clamp pulses to various potentials (ranging from -55 mV to 30 mV in 5 mV increments^1^. The density of *I_Ca,L_* in the peripheral cell model was set to -15.8 pA/pF (Figure S1 C, upper right star) following the measured peak inward current at 0 mV in response to depolarizing voltage clamp pulses to various potentials (ranging from -50 mV to 40 mV in 10 mV increments)^3^. The density of *I_Kr_* in the peripheral cell model was set to 5.8 pA/pF (Figure S1 A, upper right star) following the measured peak sensitive tail current after 1 sec pulse to -10 mV from a holding potential of -50 mV^2^. Density of *I_Ks_* in the peripheral cell model was set to 5.4 pA/pF (Figure S1 B, upper right star) following the measured peak sensitive tail current after 1 sec pulse to 40 mV from a holding potential of -50 mV^2^.

Centrally located cells are smaller than cells in the periphery^5^, and have distinct capacitances (C_m_) (central cell= 32 pF, peripheral cell= 65 pF) and lengths (central cell= 70 μm, peripheral cell= 86 μm), which are included in the model. We updated the internal Ca^2+^ clock by incorporating a recent model^6^ that is more sensitive to SR Ca^2+^ release. In the central cell model we eliminated flux of Na^+^ and K^+^ to prevent membrane potential drift. Using this updated Kurata central cell model as a base, we used experimental data to construct an updated peripheral cell model. Based on studies^7,8^ in central and peripheral cells, we fit normalized densities of *I_Ca,L_*, *I_Na_*, *I_Kr_*, *I_Ks_* in our updated peripheral cell model to match experimental data (supplemental Figure 1). We replaced the Hodgkin-Huxley *I_Na_* formulation with the Markov model for Na_V_1.5 that we previously published^9^. Finally, we increased the volume of the peripheral cell intracellular compartments for Ca^2+^ dynamics to be consistent with the increased capacitance.

In Figure S2 A, experimentally recorded APs from rabbit isolated SAN cells^10^ are shown for central (C_m_=22 pF) and peripheral (C_m_=57.5 pF) cells. Figure S2,B-C shows the simulated APs using the Zhang *et al*.^10^ models and the extended Kurata models, respectively. The extended Kurata models reproduce experimental AP morphology, excitation frequency and APD (Table S1).

***Equations for the extended version of the Kurata model.***

We replaced the Hodgkin-Huxley *I_Na_* formulation with the Markov model for Na_V_1.5 that we previously published^9^.

We updated the internal Ca^2+^ clock by incorporating a recent model^6^ that is more sensitive to SR Ca^2+^ release, below are the formulations for the added SR Ca^2+^ clock:

**Ca^2+^ release flux (j_SRCarel_) from SR via RyRs**

*j*SRCarel = *k*s·*O*·(*Ca*jSR - *Ca*sub)

*k*CaSR = *MaxSR*- (*MaxSR* - *MinSR*)/ (1 + (*EC*50_SR/*Ca*jSR)HSR)

*k*oSRCa = *k*oCa/*k*CaSR

*k*iSRCa = *k*iCa·*k*CaSR

*dR/dt* = (*k*im·*RI* - *k*iSRCa ·*Ca*sub·*R*) - (*k*oSRCa·*Ca*sub2·*R* - *k*om·*O*)

*dO/dt* =(*k*oSRCa·*Ca*sub2 ·*R* - *k*om·*O*) - (*k*iSRCa·*Ca*sub·*O* - *k*im·*I*)

*dI/dt* = (*k*iSRCa· *Ca*sub·*O* - *k*im·*I*) - (*k*om·*I* - *k*oSRCa·*Ca*sub2 ·*RI*)

*dRI/dt* = (*k*om·*I* - *k*oSRCa·*Ca*sub2·*RI*) - (*k*im·*RI* - *k*iSRCa·*Ca*sub·*R*)

**Intracellular Ca^2+^ fluxes**

*j*Ca_dif = (*Ca*sub - *Ca*i)/τdifCa

*j*up = *P*up/(1 + *K*up/*Ca*i)

*j*tr = (*Ca*nSR – *Ca*jSR)/τtr

Where *j*Ca_dif Ca^2+^ diffusion flux from submembrane space to myoplasm, *j*up Ca^2+^ uptake (pumping) by the SR and *j*tr is Ca^2+^ flux between (network and junctional) SR compartments.

**Ca^2+^ buffering**

*df*TC*/dt* = *k*fTC·*Ca*i·(1 -*f*TC) - *k*bTC · *f*TC

*df*TMC/*dt* = *k*fTMC ·*Ca*i ·(1- *f*TMC - *f*TMM) - *k*bTMC · *f*TMC

*df*TMM/*dt* = *k*fTMM ·*Mg*i ·(1-*f*TMC - *f*TMM)- *K*bTMM · *f*TMM

*df*CMi/*dt* = *k*fCM ·*Ca*i ·(1- *f*CMi) - *k*bCM · *f*CMi

*df*CMs/*dt* = *k*fCM ·*Ca*sub·(1 - *f*CMs) - *k*bCM · *f*CMs

*df*CQ/*dt* = *k*fCQ ·*Ca*jSR·(1- *f*CQ) - *k*bCQ · *f*CQ

**Dynamics of Ca^2+^ concentrations in cell compartments**

*dCa*i/*dt* =(*j*Ca_dif ·*V*sub - *j*up·*V*up) /*V*i - (*CM*tot·*df*CMi*/dt* + *TC*tot·*df*TC/*dt* + *TMC*tot·*df*TMC*/dt*)

*dCa*sub*/dt* = *j*SRCarel ·*V*jSR/*V*sub -(*I*CaL+*I*CaT+*I*bCa-2·*I*NCX)/(2·F·*V*sub)-(*j*Ca_dif + *CM*tot ·*df*CMs*/dt*)

*dCa*jSR/*dt* = *j*tr - *j*SRCarel - *CQ*tot · *df*CQ*/dt*

*dCa*nSR*/dt* = *j*up - *j*tr ·*V*jSR/*V*nSR

The 1D "non-uniform model" consists of 15 central model cells connected to 15 peripheral model cells as described above. The "uniform model" consists of 30 central model cells. Both are connected to 30 atrial cells from Lindblad et al.^11^. The lowest intercellular coupling value used in the model is 7.5 nS, which is the measured mean value in cell pairs^12^.

Following the experimental data, we assumed that cells in the center are smallest and gradually increase in size through the periphery until the border of the atria^5,13^. Cell capacitance is from 32 pF for cell #1 in the center to 65 pF for cell #30 in the periphery. The current densities in the SAN are functions of C_m_ :

Where $i$ indexes the cell position, *N* denotes the number of SAN cells, is an ion channel in the model; *c* stands for center and *p* for periphery. The subspace volume changes linearly as a function of cell length:

Where $L_{C}$ is the first central cell length (70 μm), $L_{P}$is the last peripheral cell length (86 μm) and *N* is the number of SAN cells.

Current is described by:

Where *V* is the membrane potential, *t* is time, $I_{\text{Total}}$ is the sum of the transmembrane ionic currents and is the gap-junction coupling. The 1D strand is self-pacing; in the present study, the figures are plotted from 4000 ms on (after steady-state is achieved).

***2D simulations in a 1D equivalent representation***

Experiments have shown that the sinoatrial node (SAN) is a structure with a region of small “central” cells surrounding by a region of larger “peripheral” cells^5,7,13^ and that there is a gradient in gap junction density from the center to the periphery of the SAN^14,15^. Pacemaking is initiated in the central region (in normal physiology) and spreads via the peripheral region to the atrium^7^. To reflect this geometry, we modeled the SAN as a two dimensional radially symmetric disk comprised of 30 concentric equipotential regions as shown in Figure 1A (left). Because discrete regions are assumed equipotential, the disk representation can be collapsed to a one-dimensional equivalent, where each ring is represented as a single model cell with a gradient in coupling (as a function of C_m_, see supplemental methods). The steepness of the coupling gradient in one dimension corresponds to the size of concentric rings in two–dimensions. The gradual increase in intercellular coupling is illustrated by the grayscale gradient in Figure 1, where the white center indicates low coupling and the increasingly shaded periphery indicates higher coupling.

***Leading pacemaker site***

The pacemaker is defined as the cell that first reaches excitation threshold. Threshold = time of the maximum upstroke velocity of the action potential = maximum rate of change of voltage (V_max_). Cells that reached V_max_ earliest were identified, in addition, we also followed the first peak of the 2^nd^ derivative of I_Ca,L_ (d^2^I_CaL_/dt^2^). The cells that reached V_max_ earliest and the cells that reached d^2^I_CaL_/dt^2^ were always located at the same site.

***Sensitivity of pacemaker site to changes in ionic conductances***

We tested the sensitivity of the pacemaker location to perturbations of ionic conductance values by increasing or decreasing the conductance of every ionic current by 10% to reflect variability in experimentally recorded currents. The second derivative of ICa,L for the 15th excitation was recorded for both uniform and non-uniform models. Results shown in the table indicate that pacemaker location was robust to such perturbations – central pacemaking always occurred in the uniform model and peripheral pacemaking was always present in the non-uniform model.

|  | **Pacemaker Location (cell #)** | |
| --- | --- | --- |
| **Perturbation** | uniform model | non-uniform model |
| **default** | 5 | 18 |
| **g_caL_p = 1.1 * g_caL_p** | 6 | 17 |
| **g_caL_p = 0.9 * g_caL_p** | 3 | 18 |
| **g_kr_c = 1.1 * g_kr_c** | 3 | 17 |
| **g_kr_c = 0.9 * g_kr_c** | 6 | 18 |
| **g_kr_p = 1.1 * g_kr_p** | 6 | 17 |
| **g_kr_p = 0.9 * g_kr_p** | 3 | 18 |
| **g_st_c = 1.1 * g_st_c** | 2 | 17 |
| **g_st_c = 0.9 * g_st_c** | 7 | 18 |
| **g_st_p = 1.1 * g_st_p** | 7 | 18 |
| **g_st_p = 0.9 * g_st_p** | 1 | 17 |
| **g_caT_c = 1.1 * g_caT_c** | 2 | 17 |
| **g_caT_c = 0.9 * g_caT_c** | 7 | 18 |
| **g_caT_p = 1.1 * g_caT_p** | 8 | 18 |
| **g_caT_p = 0.9 * g_caT_p** | 1 | 17 |
| **g_ks_c = 1.1 * g_ks_c** | 5 | 18 |
| **g_ks_c = 0.9 * g_ks_c** | 5 | 18 |
| **g_ks_p = 1.1 * g_ks_p** | 5 | 18 |
| **g_ks_p = 0.9 * g_ks_p** | 5 | 17 |
| **g_to_c = 1.1 * g_to_c** | 5 | 18 |
| **g_to_c = 0.9 * g_to_c** | 4 | 18 |
| **g_to_p = 1.1 * g_to_p** | 4 | 18 |
| **g_to_p = 0.9 * g_to_p** | 5 | 17 |
| **g_sus_c = 1.1 * g_sus_c** | 4 | 17 |
| **g_sus_c = 0.9 * g_sus_c** | 6 | 18 |
| **g_sus_p = 1.1 * g_sus_p** | 6 | 18 |
| **g_sus_p = 0.9 * g_sus_p** | 4 | 17 |
| **g_h_c = 1.1 * g_h_c** | 5 | 18 |
| **g_h_c = 0.9 * g_h_c** | 5 | 18 |
| **g_h_p = 1.1 * g_h_p** | 5 | 18 |
| **g_h_p = 0.9 * g_h_p** | 5 | 18 |
| **g_na_c = 1.1 * g_na_c** | 5 | 18 |
| **g_na_c = 0.9 * g_na_c** | 5 | 18 |
| **g_na_p = 1.1 * g_na_p** | 5 | 18 |
| **g_na_p = 0.9 * g_na_p** | 5 | 17 |
| **g_bna_c = 1.1 * g_bna_c** | 3 | 17 |
| **g_bna_c = 0.9 * g_bna_c** | 6 | 18 |
| **g_bna_p = 1.1 * g_bna_p** | 6 | 19 |
| **g_bna_p = 0.9 * g_bna_p** | 3 | 16 |
| **g_kAch_c = 1.1 * g_kAch_c** | 6 | 18 |
| **g_kAch_c = 0.9 * g_kAch_c** | 4 | 17 |
| **g_kAch_p = 1.1 * g_kAch_p** | 4 | 17 |
| **g_kAch_p = 0.9 * g_kAch_p** | 6 | 18 |
| **i_naKmax_c = 1.1 * i_naKmax_c** | 6 | 18 |
| **i_naKmax_c = 0.9 * i_naKmax_c** | 2 | 17 |
| **i_naKmax_p = 1.1 * i_naKmax_p** | 2 | 17 |
| **i_naKmax_p = 0.9 * i_naKmax_p** | 7 | 18 |
| **k_naca_c = 1.1 * k_naca_c** | 3 | 18 |
| **k_naca_c = 0.9 * k_naca_c** | 6 | 17 |
| **k_naca_p = 1.1 * k_naca_p** | 6 | 17 |
| **k_naca_p = 0.9 * k_naca_p** | 3 | 18 |

***AP properties***

MDP (mV) is the most negative diastolic membrane potential. UV = d*V*/d*t*_max_, V/s. CL (ms) is the interval between MDPs. AP duration (APD, ms) is time between d*V*/d*t*_max_ and 90% cellular repolarization (APD90).

Take-off potential (TOP, mV) was defined at the time when (d*V*/d*t*) = 0.5mV/ms. The slope of diastolic depolarization (DD, mV/ms) is the slope of the voltage between MDP and TOP. The SAN-atrium conduction time (SACT) is the time between d*V*/d*t*_max_ of the leading pacemaker and the first atrial cell.

***Simulation of vagal stimulation***

As we have done previously ^16^, we increased background inward rectifier current 4x. This was sufficient to hyperpolarize the maximum diastolic potential (MDP) 10 mV, approximating what has been reported experimentally with high concentrations of acetylcholine (ACh) ^17^ ^7^ ^18^. In addition, we included a 10 mV negative shift of pacemaker current (I_f_) activation and a 10% reduction of L-type Ca^2+^ current amplitude to mimic effects of strong vagal stimulation.

***3D simulations in a 2D equivalent representation***

An SAN in three dimensions is constructed by stacking thirty 2D disks (described above) flanked by rings comprised of atrial model cells as shown in Figure 4. Since the mathematical equivalent of the 3D structure is 2D slice with discrete rows that are assumed equipotential, we ran simulations in the simpler slice model, where each ring is represented as a single row with a gradient in coupling (as a function of C_m_). The steepness of the coupling gradient in two dimensions corresponds to the size of concentric rings in three–dimensions.

The ionic current densities were functions of C_m_:

Where $i$ indexes x-coordinates and$j$ indexes y-coordinates, *N* = number of SAN cells, = ion channel; *c* = center and *p* = periphery.

***Simulations incorporating a model of atrial fibroblasts***

The fibroblast model consists of a membrane capacitance and an ohmic resistance connected in parallel. Membrane capacitance is 18 pF based on experimental observations in atrial fibroblasts^19^. Intercellular coupling is set to either 0 nS (uncoupled) 1 nS, 3 nS and 6 nS (coupled).

To study the influence of fibroblasts on impulse propagation, we used the tissue representation described above and shown in Figure 4. As has been observed experimentally *in vivo* in the rabbit SAN, fibroblasts are found either interspersed with pacemaker myocytes or as islands of connective tissue^20^. Using these data as a guide, we incorporated 5 different fibroblast island distributions within the SAN constrained by the assumption that no island borders another island. These distributions, represented by 30x30 matrices, differ in the number of fibroblasts per island (represented as 2 x 2, 4 x 4 and 6 x 6 model fibroblast cell arrays) and by their fibroblast density (10%, 25% and 50% tissue coverage). The fibroblasts and their neighboring myocytes or fibroblasts were electrically coupled with a G_Gap_ varying from 0-6 nS (0 nS for uncoupled fibroblasts and 1 nS, 3 nS and 6 nS for fibroblasts coupled to myocytes or fibroblasts). This range was chosen because it reflects the values ranging from 0.31 to 8 nS measured, estimated or inferred from cell culture experiments^21-24^.

**Supplemental Figures:**

Figure S1: Correlation between the density of ionic currents and the size of rabbit SAN cells in experiments and in our updated Kurata central and peripheral models (red star). Densities of *I_Kr_* (A), *I_Ks_* (B) *I_Ca,L_* (C) and *I_Na_* (D) are plotted against C_m_. Data from Lei et al.^2^ (*I_Kr_*, *I_Ks_*) Musa et al.^3^ (*I_Ca,L_*) and Honjo et al.^1^ (*I_Na_*).

Table 1:

Listing of experimental data^25^ parameters compared with the Zhang et al.^10^ and the updated Kurata (present model) cell models.

Figure 2:

Simulated central and peripheral SAN APs. A) Action potentials recorded from rabbit isolated SAN cells with Cm of 22 pF and 57.5 pF^10^. B) Simulated central and peripheral APs using Zhang et al.^10^ and C) updated Kurata models.

Figure 3:

The 5 distributions for 2 x 2, 4 x 4 and 6 x 6 sized fibroblast islands with fibroblast density as indicated.

Figure 4:

The updated uniform model simulates accurately observed properties of the intact SAN. Model simulations (left) compared to experiments. Note the gradual change in AP morphology and UV beginning from center (bottom arrow) to periphery and to atria (top arrow).

Figure 5:

A simulation showing the effects of vagal stimulation in the central region (first 15 cells) of the mosaic model is shown in Supplemental Figure S5. Consistent with our previous results, vagal stimulation in the mosaic model results in a peripheral shift of pacemaking and a slowing in the heart rate to 425 ms CL (~31%).

**Figure 1:**


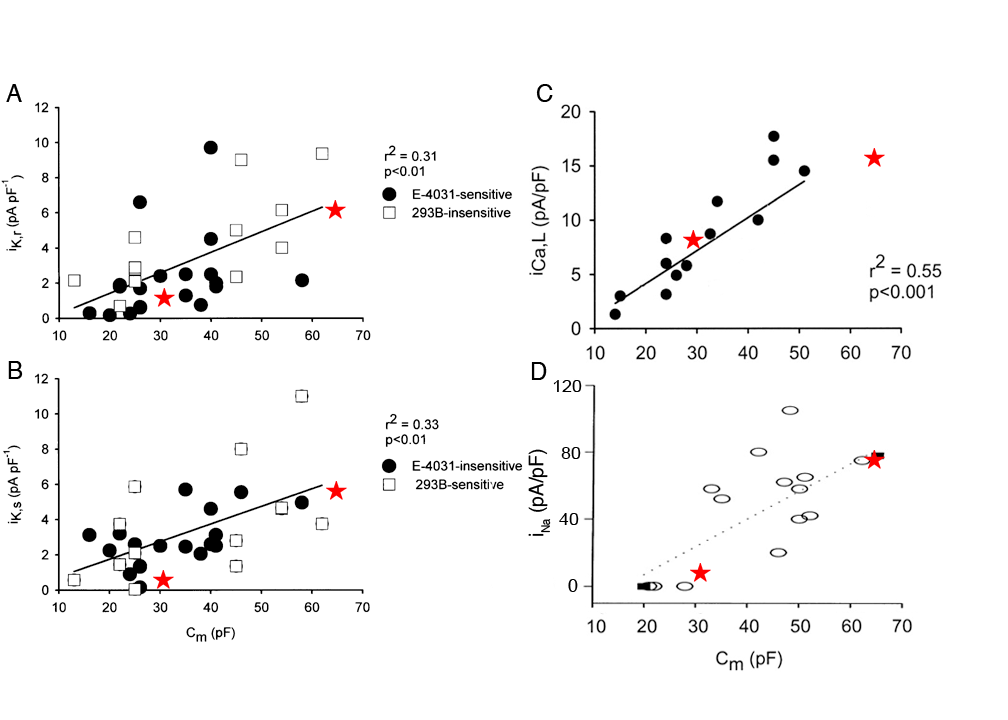


**Table 1:**

|  | MDP (mV) | POP (mV) | CL (ms) | UV (V/s) |
| --- | --- | --- | --- | --- |
| Kodama et al. (center)^25^ | -53 | 15 | 350 | 1.6 |
| Kodama et al. (periphery)^25^ | -70 | 28 | 170 | 60 |
| Zhang et al (center)^10^ | -56.2 | 19.5 | 325 | 2.4 |
| Zhang et al (periphery)^10^ | -77.8 | 24.5 | 160.4 | 61.7 |
| Present model (center) | -56.1 | 16.6 | 301 | 6.0 |
| Present model (periphery) | -75.0 | 28.6 | 212.7 | 58.7 |

**Figure 2:**

**
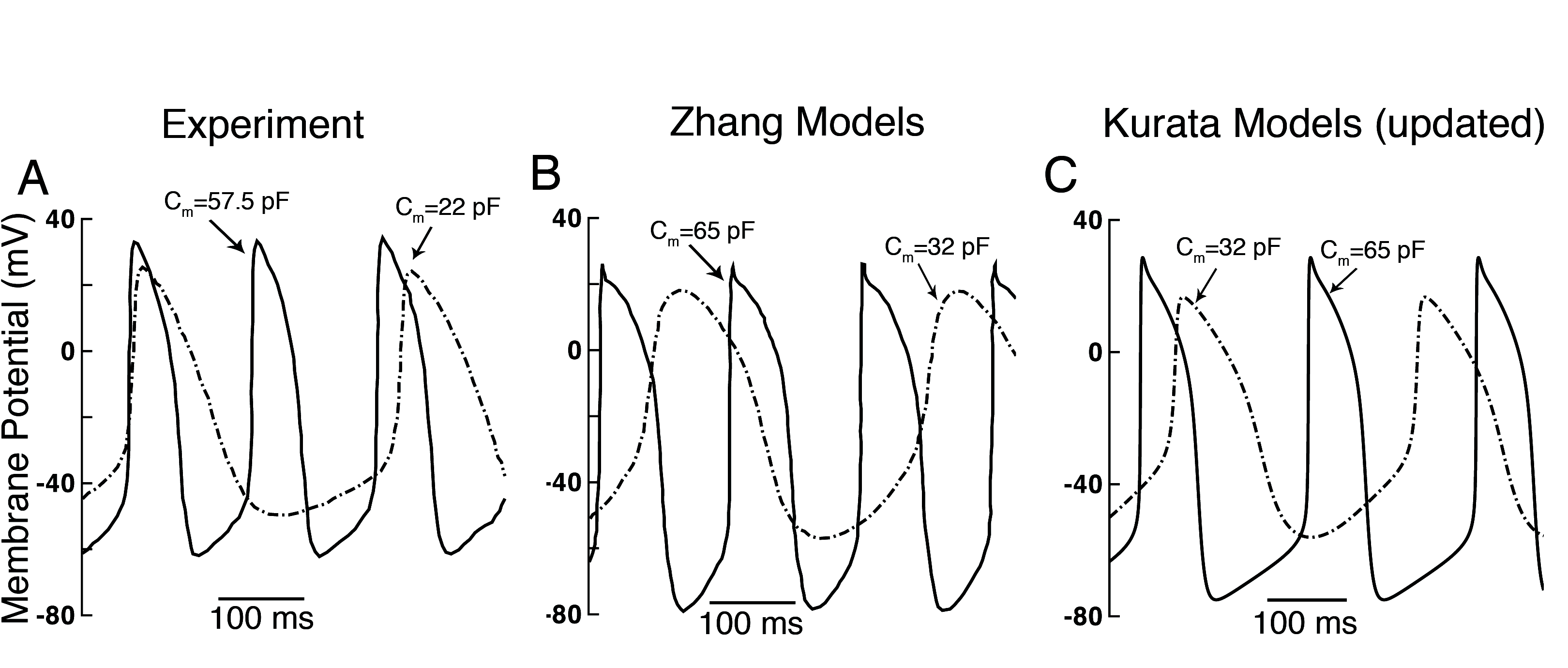
**

**Figure 3:**


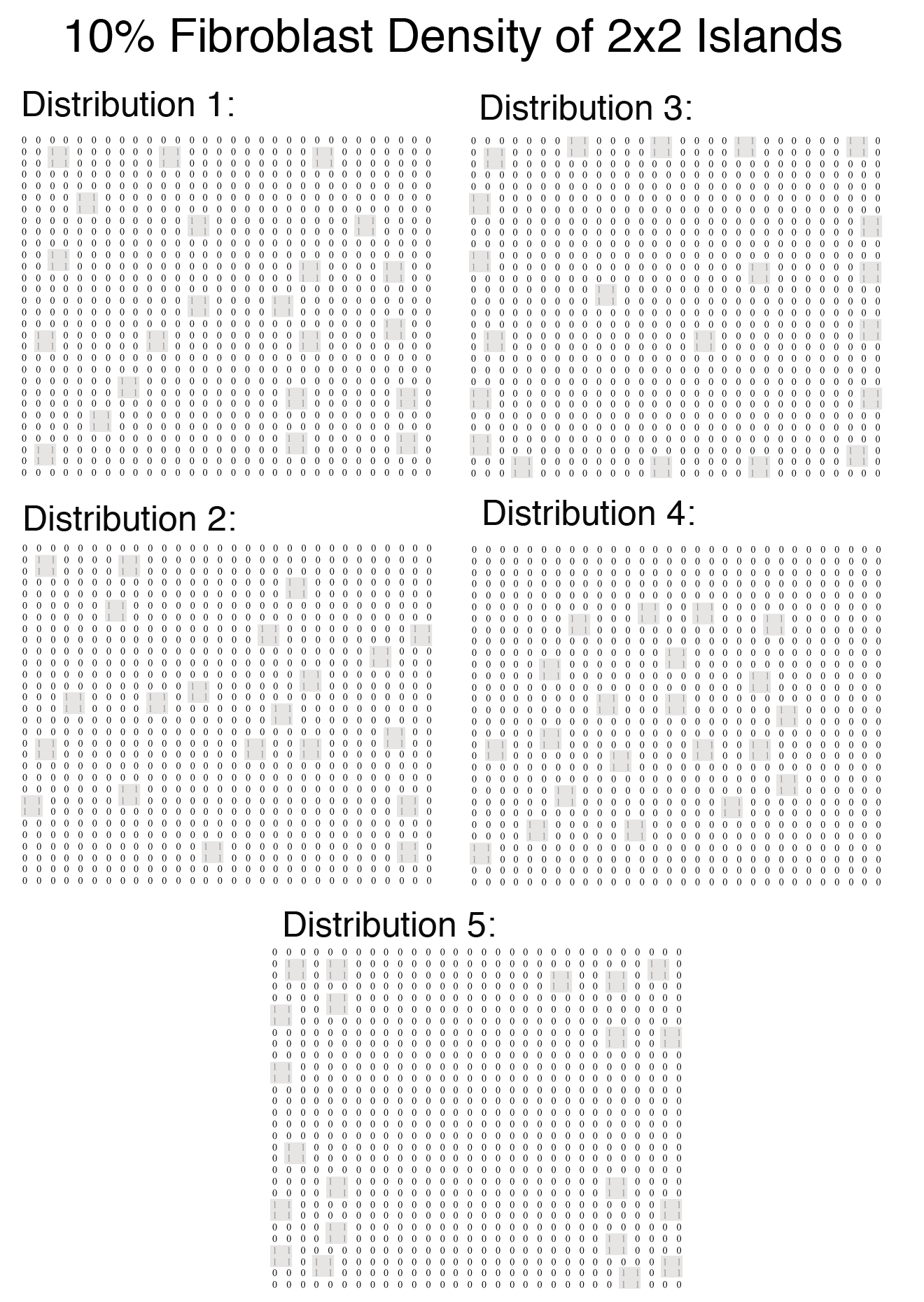


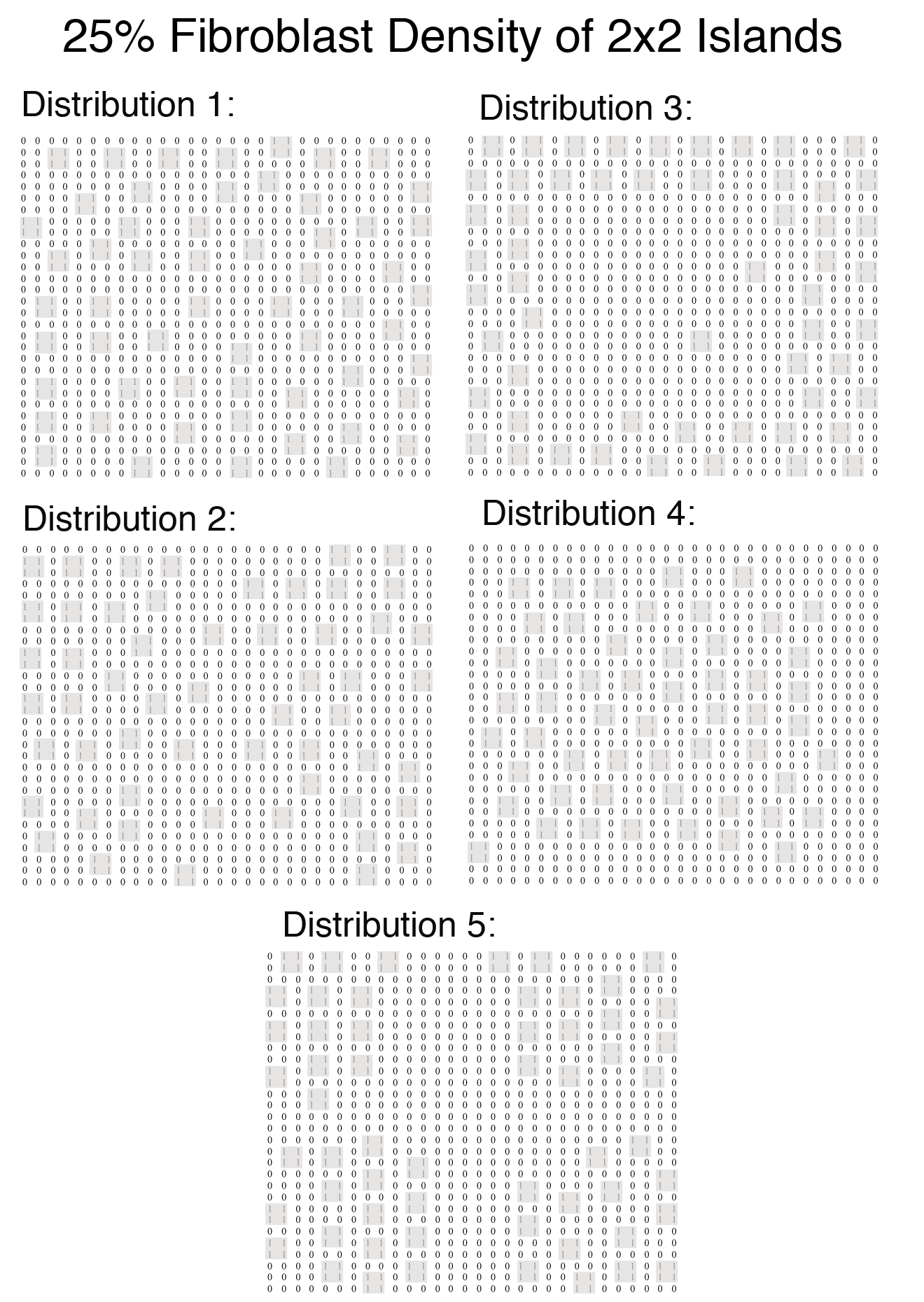


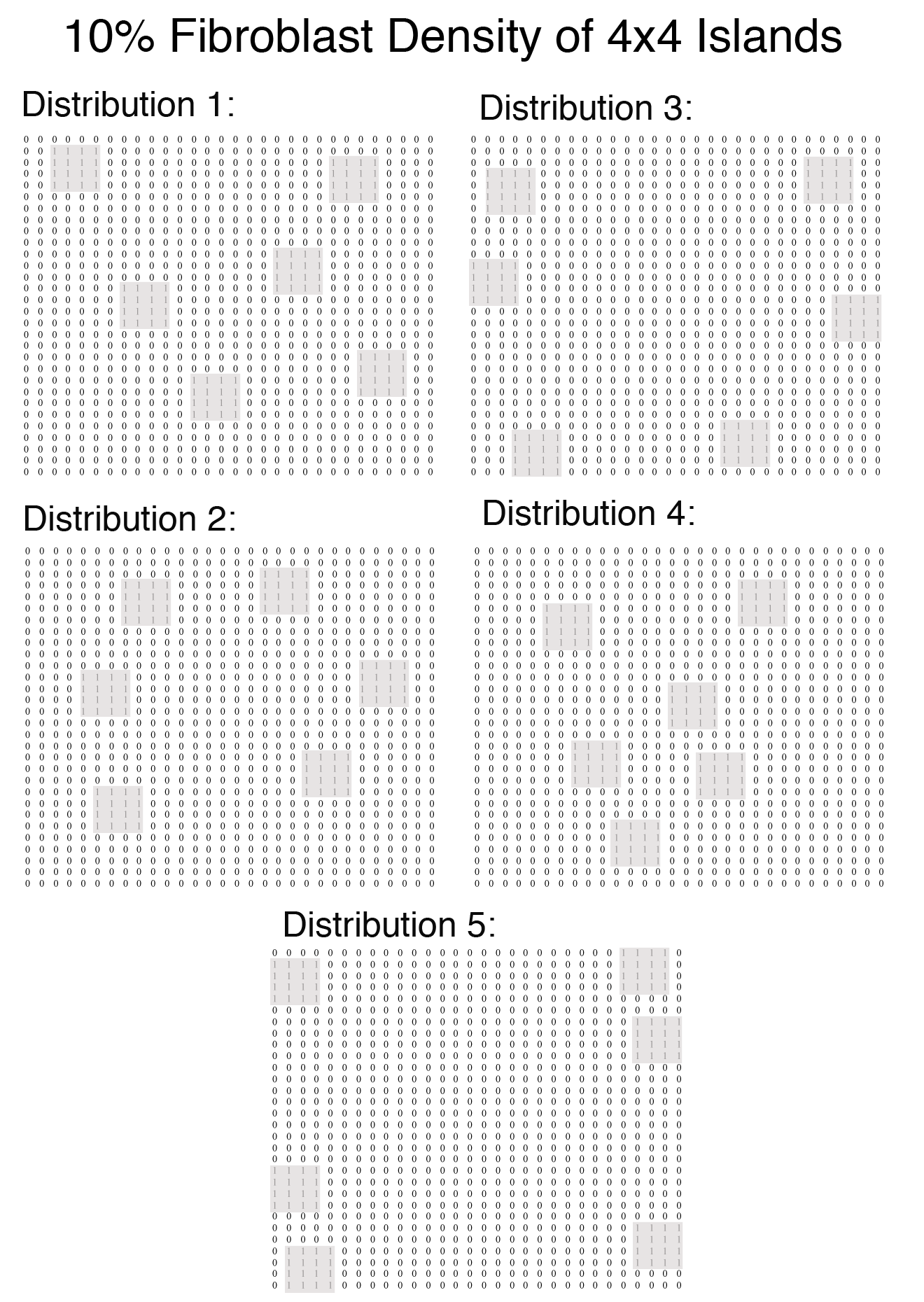


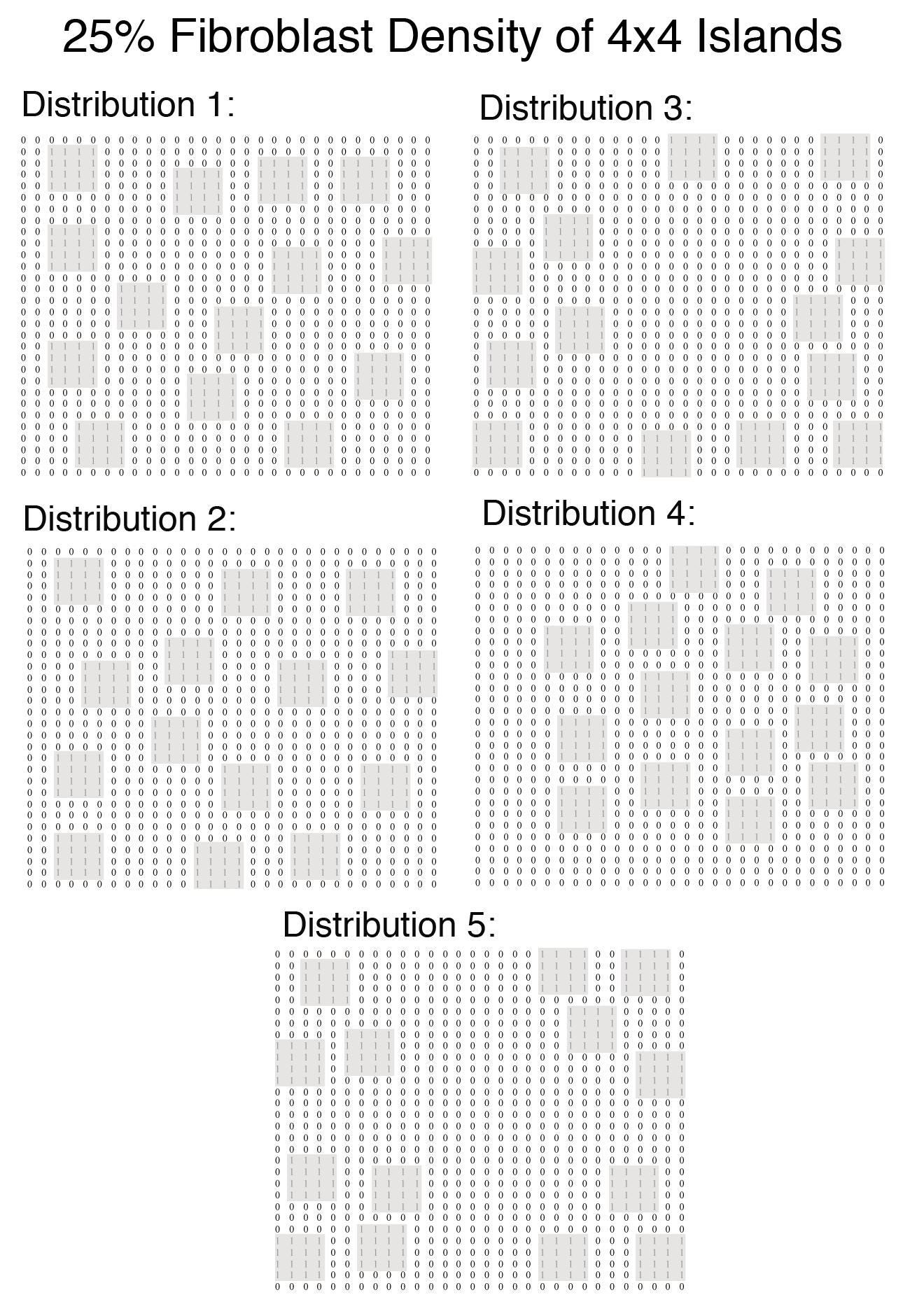


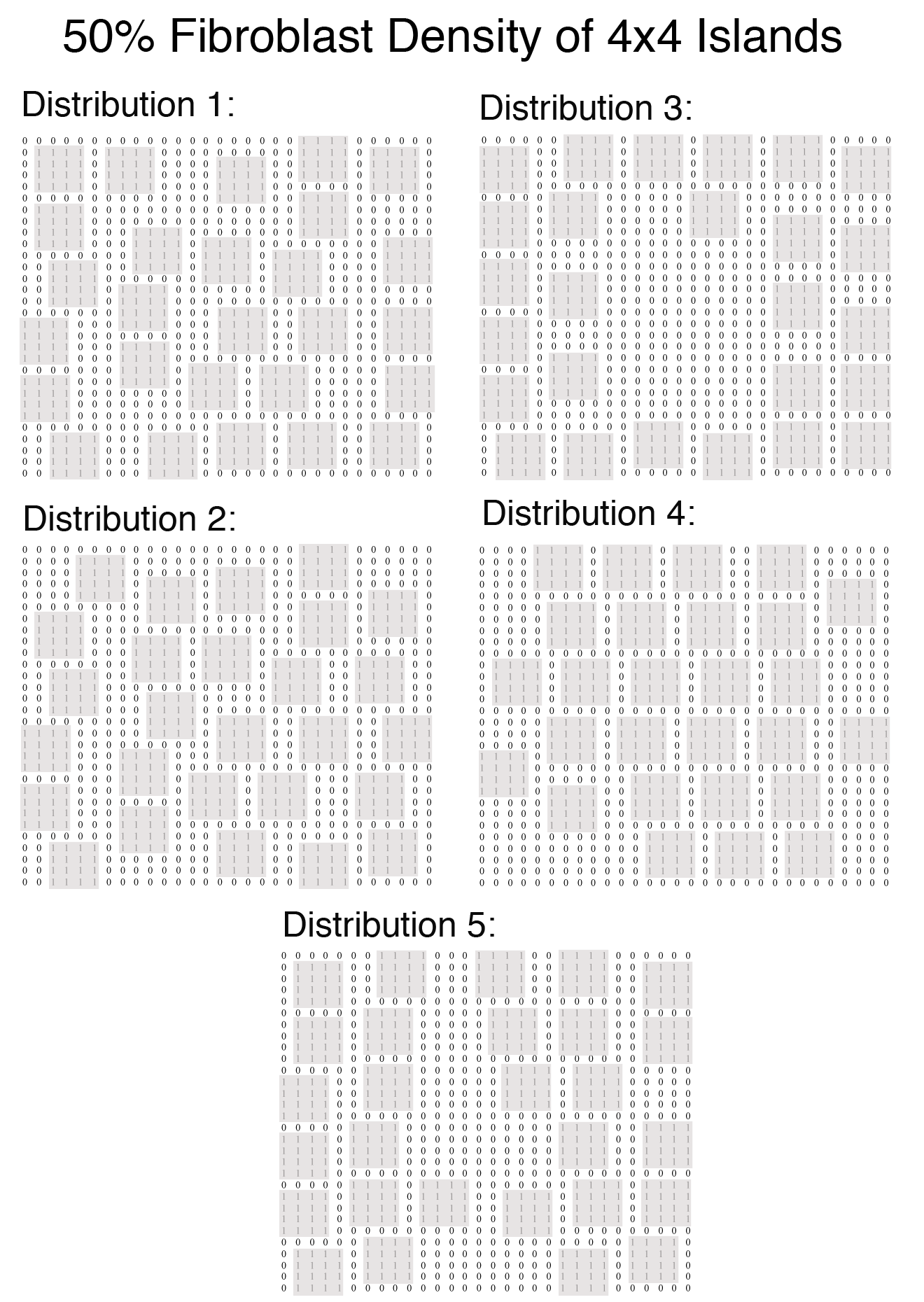


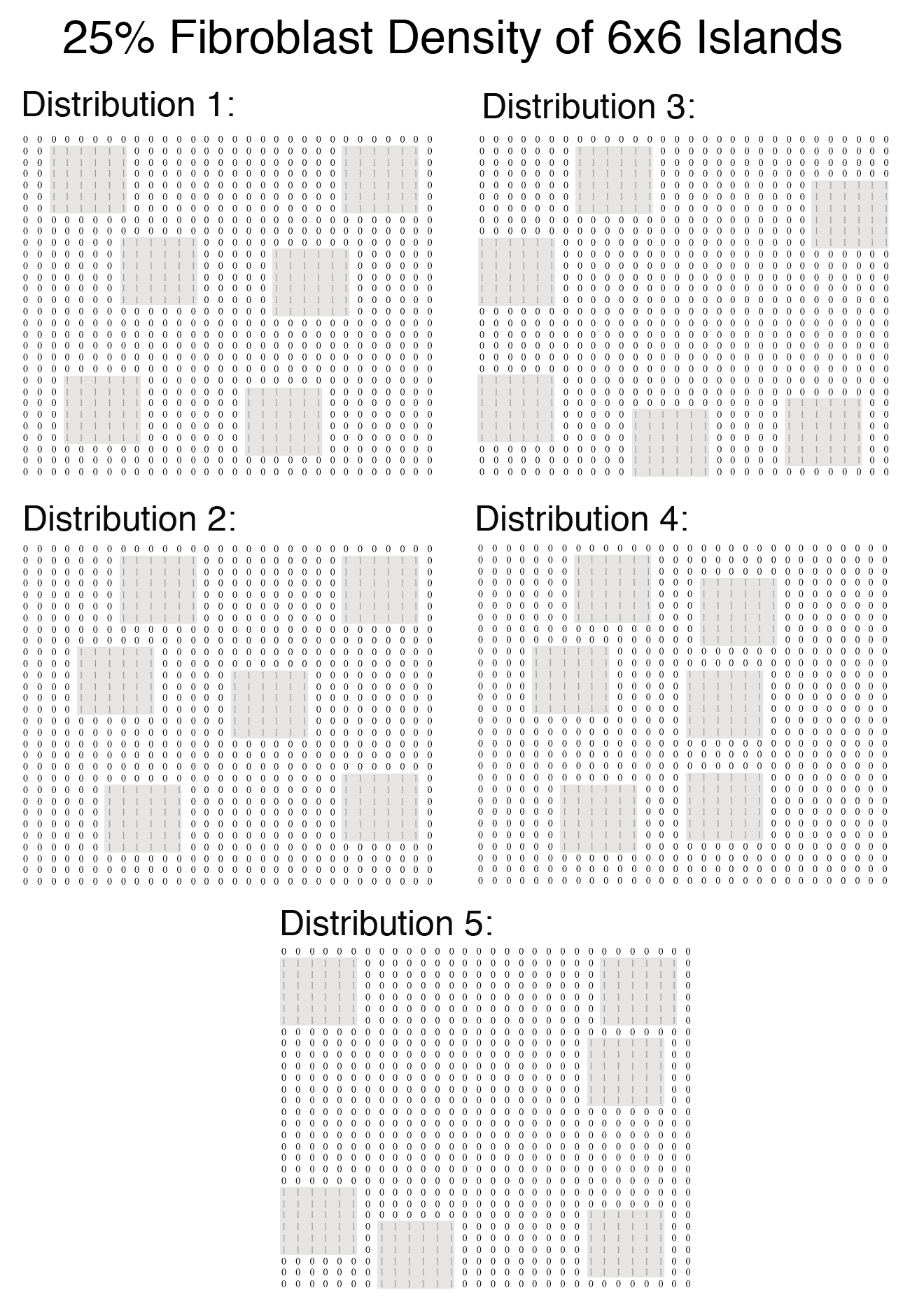


**
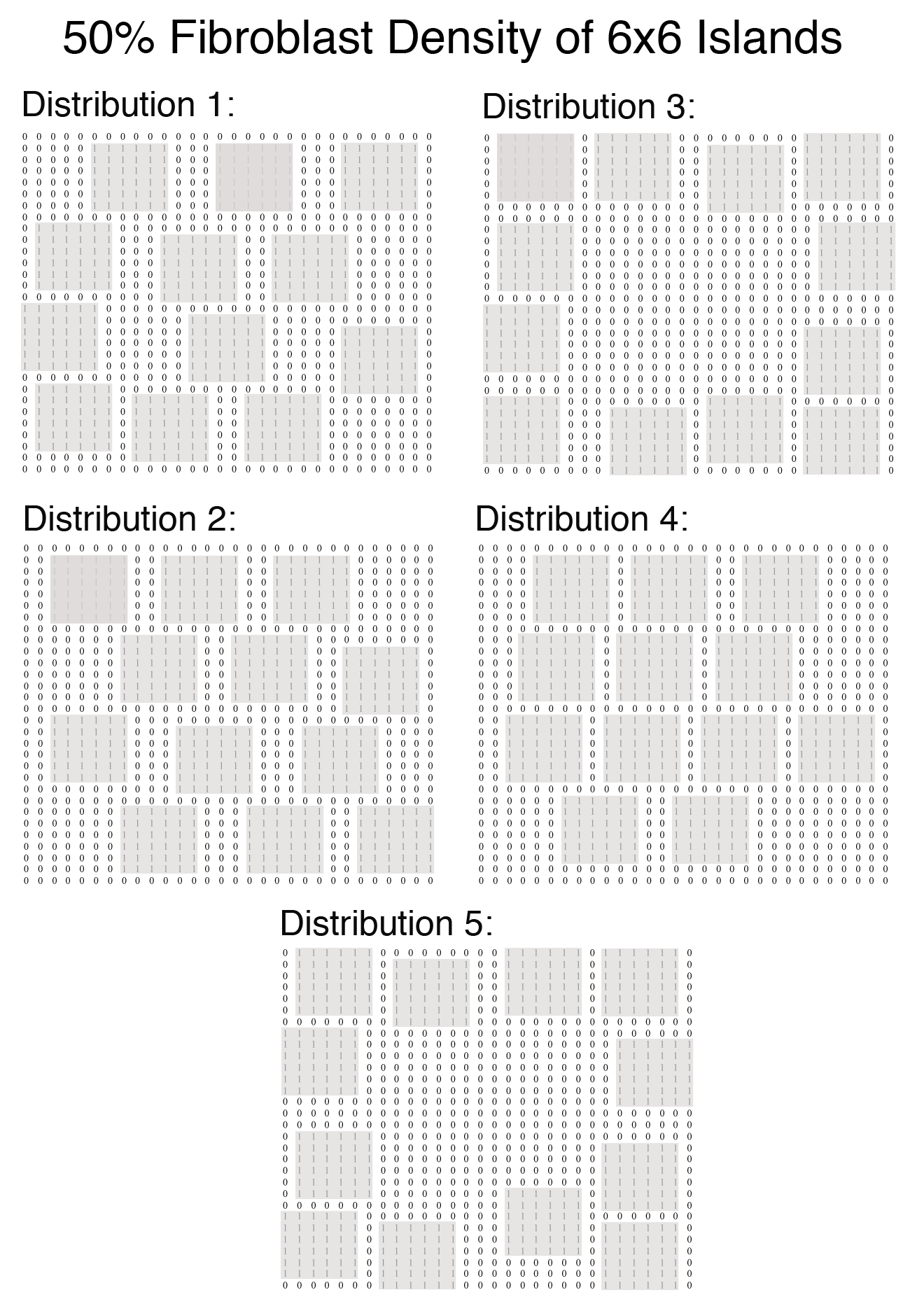
**

**Figure 4:**

**
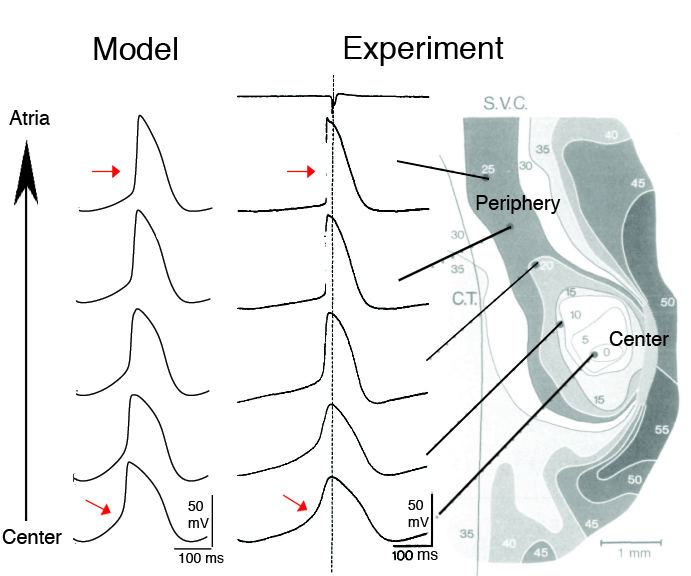
**

**Figure 5:**

**
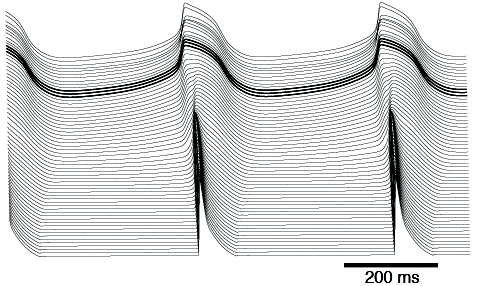
**

|  | Model Values  **SAN only SAN mosaic** | Experimental Values |
| --- | --- | --- |
| CL, ms | 301 322 | 361 ± 38 ^(37)^ |
| SACT, ms | 13.9 34.6 | 21 ± 11 ^(37)^ |
| MDP (central SAN), mV | -57.6 -63.6 | -56 ± 11 ^(37)^ |
| MDP (peripheral SAN), mV | -68.6 -73 | -61 ± 6 ^(37)^ |
| APD90 (central SAN), ms | 142.9 137.3 | 120-150 ^(2)^ |
| APD90 (peripheral SAN), ms | 107 112.8 | 80-120 ^(2)^ |
| dv/dt max (central SAN), V/s | 6.1 4.2 | <10 ^(2)^ |
| dv/dt max (peripheral SAN), V/s | 13.8 18 | 10-50 ^(2)^ |

**Table 2:** *Summary of measured parameters in the intact SAN model and experimentally observed values*.

**References:**

1 Honjo, H., Boyett, M. R., Kodama, I. & Toyama, J. Correlation between electrical activity and the size of rabbit sino-atrial node cells. *J Physiol* **496 ( Pt 3)**, 795-808 (1996).

2 Lei, M., Honjo, H., Kodama, I. & Boyett, M. R. Heterogeneous expression of the delayed-rectifier K+ currents i(K,r) and i(K,s) in rabbit sinoatrial node cells. *J Physiol* **535**, 703-714 (2001).

3 Musa, H. *et al.* Heterogeneous expression of Ca(2+) handling proteins in rabbit sinoatrial node. *J Histochem Cytochem* **50**, 311-324 (2002).

4 Kurata, Y., Hisatome, I., Imanishi, S. & Shibamoto, T. Dynamical description of sinoatrial node pacemaking: improved mathematical model for primary pacemaker cell. *Am J Physiol Heart Circ Physiol* **283**, H2074-2101 (2002).

5 Dobrzynski, H. *et al.* Computer three-dimensional reconstruction of the sinoatrial node. *Circulation* **111**, 846-854 (2005).

6 Maltsev, V. A. & Lakatta, E. G. Synergism of coupled subsarcolemmal Ca2+ clocks and sarcolemmal voltage clocks confers robust and flexible pacemaker function in a novel pacemaker cell model. *Am J Physiol Heart Circ Physiol* **296**, H594-615, doi:01118.2008 [pii]10.1152/ajpheart.01118.2008 (2009).

7 Boyett, M. R., Honjo, H. & Kodama, I. The sinoatrial node, a heterogeneous pacemaker structure. *Cardiovasc Res* **47**, 658-687 (2000).

8 Boyett, M. R. *et al.* The sinoatrial node: cell size does matter. *Circ Res* **101**, e81-82 (2007).

9 Clancy, C. E., Tateyama, M. & Kass, R. S. Insights into the molecular mechanisms of bradycardia-triggered arrhythmias in long QT-3 syndrome. *J Clin Invest* **110**, 1251-1262 (2002).

10 Zhang, H. *et al.* Mathematical models of action potentials in the periphery and center of the rabbit sinoatrial node. *Am J Physiol Heart Circ Physiol* **279**, H397-421 (2000).

11 Lindblad, D. S., Murphey, C. R., Clark, J. W. & Giles, W. R. A model of the action potential and underlying membrane currents in a rabbit atrial cell. *Am J Physiol* **271**, H1666-1696 (1996).

12 Verheule, S., van Kempen, M. J., Postma, S., Rook, M. B. & Jongsma, H. J. Gap junctions in the rabbit sinoatrial node. *Am J Physiol Heart Circ Physiol* **280**, H2103-2115 (2001).

13 Opthof, T., de Jonge, B., Jongsma, H. J. & Bouman, L. N. Functional morphology of the mammalian sinuatrial node. *Eur Heart J* **8**, 1249-1259 (1987).

14 Bleeker, W. K., Mackaay, A. J., Masson-Pevet, M., Bouman, L. N. & Becker, A. E. Functional and morphological organization of the rabbit sinus node. *Circ Res* **46**, 11-22 (1980).

15 Masson-Pevet, M., Bleeker, W. K. & Gros, D. The plasma membrane of leading pacemaker cells in the rabbit sinus node. A qualitative and quantitative ultrastructural analysis. *Circ Res* **45**, 621-629 (1979).

16 Protas, L., Oren, R. V., Clancy, C. E. & Robinson, R. B. Age-dependent changes in Na current magnitude and TTX-sensitivity in the canine sinoatrial node. *J Mol Cell Cardiol* **48**, 172-180, doi:S0022-2828(09)00320-4 [pii] 10.1016/j.yjmcc.2009.07.028 (2010).

17 Boyett, M. R. *et al.* Ionic basis of the chronotropic effect of acetylcholine on the rabbit sinoatrial node. *Cardiovasc Res* **29**, 867-878 (1995).

18 DiFrancesco, D. The pacemaker current (I(f)) plays an important role in regulating SA node pacemaker activity. *Cardiovasc Res* **30**, 307-308 (1995).

19 Kamkin, A., Kiseleva, I. & Isenberg, G. Activation and inactivation of a non-selective cation conductance by local mechanical deformation of acutely isolated cardiac fibroblasts. *Cardiovasc Res* **57**, 793-803 (2003).

20 Camelliti, P., Green, C. R., LeGrice, I. & Kohl, P. Fibroblast network in rabbit sinoatrial node: structural and functional identification of homogeneous and heterogeneous cell coupling. *Circ Res* **94**, 828-835 (2004).

21 Kamkin, A., Kiseleva, I., Lozinsky, I. & Scholz, H. Electrical interaction of mechanosensitive fibroblasts and myocytes in the heart. *Basic Res Cardiol* **100**, 337-345, doi:10.1007/s00395-005-0529-4 (2005).

22 Kawara, T. *et al.* Activation delay after premature stimulation in chronically diseased human myocardium relates to the architecture of interstitial fibrosis. *Circulation* **104**, 3069-3075 (2001).

23 Miragoli, M., Gaudesius, G. & Rohr, S. Electrotonic modulation of cardiac impulse conduction by myofibroblasts. *Circ Res* **98**, 801-810, doi:01.RES.0000214537.44195.a3 [pii]

10.1161/01.RES.0000214537.44195.a3 (2006).

24 Rook, M. B. *et al.* Differences in gap junction channels between cardiac myocytes, fibroblasts, and heterologous pairs. *Am J Physiol* **263**, C959-977 (1992).

25 Kodama, I. *et al.* Regional differences in the role of the Ca2+ and Na+ currents in pacemaker activity in the sinoatrial node. *Am J Physiol* **272**, H2793-2806 (1997).
